# Development and validation of a copper-related gene prognostic signature in hepatocellular carcinoma

**DOI:** 10.3389/fcell.2023.1157841

**Published:** 2023-07-18

**Authors:** Haoting Shi, Jingxuan Huang, Xue Wang, Runchuan Li, Yiqing Shen, Bowen Jiang, Jinjun Ran, Rong Cai, Fang Guo, Yufei Wang, Gang Ren

**Affiliations:** ^1^ Department of Radiation Therapy, Ruijin Hospital, Shanghai Jiao Tong University School of Medicine, Shanghai, China; ^2^ Department of Clinical Medicine, Shanghai Jiao Tong University School of Medicine, Shanghai, China; ^3^ Department of Pathology, Ruijin Hospital, Shanghai Jiao Tong University School of Medicine, Shanghai, China; ^4^ Department of Computer Science, Johns Hopkins University, Baltimore, MD, United States; ^5^ College of Biophotonics, South China Normal University, Guangzhou, China; ^6^ School of Public Health, Shanghai Jiao Tong University School of Medicine, Shanghai, China; ^7^ School of Public Health, Li Ka Shing Faculty of Medicine, The University of Hong Kong, Hong Kong, Hong Kong SAR, China; ^8^ Department of Radiology, Xinhua Hospital, Shanghai Jiao Tong University School of Medicine, Shanghai, China

**Keywords:** liver cancer, cuproptosis, hepatocellular carcinoma, copper, gene

## Abstract

**Introduction:** Reliable biomarkers are in need to predict the prognosis of hepatocellular carcinoma (HCC). Whilst recent evidence has established the critical role of copper homeostasis in tumor growth and progression, no previous studies have dealt with the copper-related genes (CRGs) signature with prognostic potential in HCC.

**Methods:** To develop and validate a CRGs prognostic signature for HCC, we retrospectively included 353 and 142 patients as the development and validation cohort, respectively. Copper-related Prognostic Signature (Copper-PSHC) was developed using differentially expressed CRGs with prognostic value. The hazard ratio (HR) and the area under the time-dependent receiver operating characteristic curve (AUC) during 3-year follow-up were utilized to evaluate the performance. Additionally, the Copper-PSHC was combined with age, sex, and cancer stage to construct a Copper-clinical-related Prognostic Signature (Copper-CPSHC), by multivariate Cox regression. We further explored the underlying mechanism of Copper-PSHC by analyzing the somatic mutation, functional enrichment, and tumor microenvironment. Potential drugs for the high-risk group were screened.

**Results:** The Copper-PSHC was constructed with nine CRGs. Patients in the high-risk group demonstrated a significantly reduced overall survival (OS) (adjusted HR, 2.65 [95% CI, 1.83–3.84] and 3.30, [95% CI, 1.27–8.60] in the development and validation cohort, respectively). The Copper-PSHC achieved a 3-year AUC of 0.74 [95% CI, 0.67–0.82] and 0.71 [95% CI, 0.56–0.86] for OS in the development and validation cohort, respectively. Copper-CPSHC yield a 3-year AUC of 0.73 [95% CI, 0.66–0.80] and 0.72 [95% CI, 0.56–0.87] for OS in the development and validation cohort, respectively. Higher tumor mutation burden, downregulated metabolic processes, hypoxia status and infiltrated stroma cells were found for the high-risk group. Six small molecular drugs were screened for the treatment of the high-risk group.

**Conclusion:** Copper-PSHC services as a promising tool to identify HCC with poor prognosis and to improve disease outcomes by providing potential clinical decision support in treatment.

## Introduction

Liver cancer is the second leading cause of cancer-related death and the seventh most common cancer worldwide (Sung et al., 2021). Hepatocellular carcinoma (HCC) is unequivocally the most dominant type of liver cancer, accounting for 90% of all cases (Llovet et al., 2021). The disease burden of HCC has been rising, with over 1 million new cases per year being estimated during the next decade globally (Llovet et al., 2018). Despite recent advances in the clinical management of HCC including both local and systemic therapies, there remain large and growing unmet medical needs (Villanueva, 2019). Due to occult onset and limited treatment efficacy, HCC is generally subject to poor prognosis (Golabi et al., 2017), with the 5-year survival rate as low as 18% in the United States (Jemal et al., 2017). The conventional clinical decisions for HCC treatment depend substantially on the tumor stage employing the Barcelona Clinic Liver Cancer (BCLC) staging system (EASL Clinical Practice Guidelines, 2018) and Tumor Node Metastasis (TNM) staging system. However, these staging systems, which take primarily tumor size and metastasis into account, have failed to benefit a considerable proportion of patients, owing to their insensitivity to the molecular features in HCC (Yang et al., 2019). Thus, it is crucial to formulate a more precise and Supplementary Model to identify the segments of HCC patients who are at high risk of unfavorable prognosis necessitating additional treatment or targeted therapy (Solimando et al., 2022), so as to improve the survival rate and terminal life quality of patients with better clinical decision making. With the insight into the biology of HCC updated, several biomarkers and gene expression-based signatures have been proposed (Mann et al., 2007; van Malenstein et al., 2011; Wu et al., 2020; Dai et al., 2021); yet, they were rarely incorporated into clinical practice due to less-than-satisfactory performance and insufficient validation (Liu et al., 2019), which warrants a high demand for novel and robust prognostic models.

Elevated levels of copper have been previously observed in the malignant neoplasms of breast, lung, and gastrointestinal tract (Jin et al., 2011; Adeoti et al., 2015; Stepien et al., 2017), indicating an essential role of copper in the genesis of carcinoma. Specifically, increased cellular copper concentrations might contribute to cancer progression by enhancing blood vessel formation which is critical for tumor initiation, growth and metastasis (Blockhuys et al., 2017). With the concept of “Cuproplasia” (i.e., copper-dependent cell growth and proliferation) being proposed, the diverse mechanisms of copper sensing involved in the cancer have been further unveiled (Ishida et al., 2013). Meanwhile, cuproptosis, a newly proposed form of cell death triggered by copper overloads (Tsvetkov et al., 2022), was found to be closely linked to cancer such as clear cell renal cell carcinoma (Bian et al., 2022). Notably, as the central regulatory organ of copper homeostasis, the liver is particularly susceptible to copper-related carcinogenesis (Kim et al., 2008). Patients with Wilson’s disease, characterized by a progressively increased copper load in the liver, are more likely to develop liver cancer than the general population (Bandmann et al., 2015). This finding indicates that elevated intracellular copper levels would impair the liver physiological functions and increase the risk of developing HCC (McGlynn et al., 2021). Additionally, serum copper concentrations were demonstrated to be correlated with the BCLC stage (Tamai et al., 2020). The alterations in copper transporter genes, such as *ATP7A*, *ATP7B*, *SLC31A1*, and *SLC31A2*, were also found to be associated with poor prognosis in HCC patients (Davis et al., 2020). Those findings collectively highlight an important role of copper in the HCC, suggesting that copper-related biomarkers might provide valuable information for the treatment and prognosis of HCC.

Copper-related genes (CRGs) which regulate copper metabolisms including copper homeostasis, cuproptosis and copper binding (Ge et al., 2022) serve as a valid channel for us to examine the copper-HCC link. Hence, in this study, we used publicly available gene dataset to develop a prognostic stratification model, Copper-related Prognostic Signature (Copper-PSHC), for HCC patients based on CRGs. We then incorporated Copper-PSHC with clinical factors to establish an integrative prognostic model for pragmatic application. Beyond that, we also explored the potential underlying mechanism of Copper-PSHC.

## Materials and method

### Study design and patients

**FIGURE 1:**
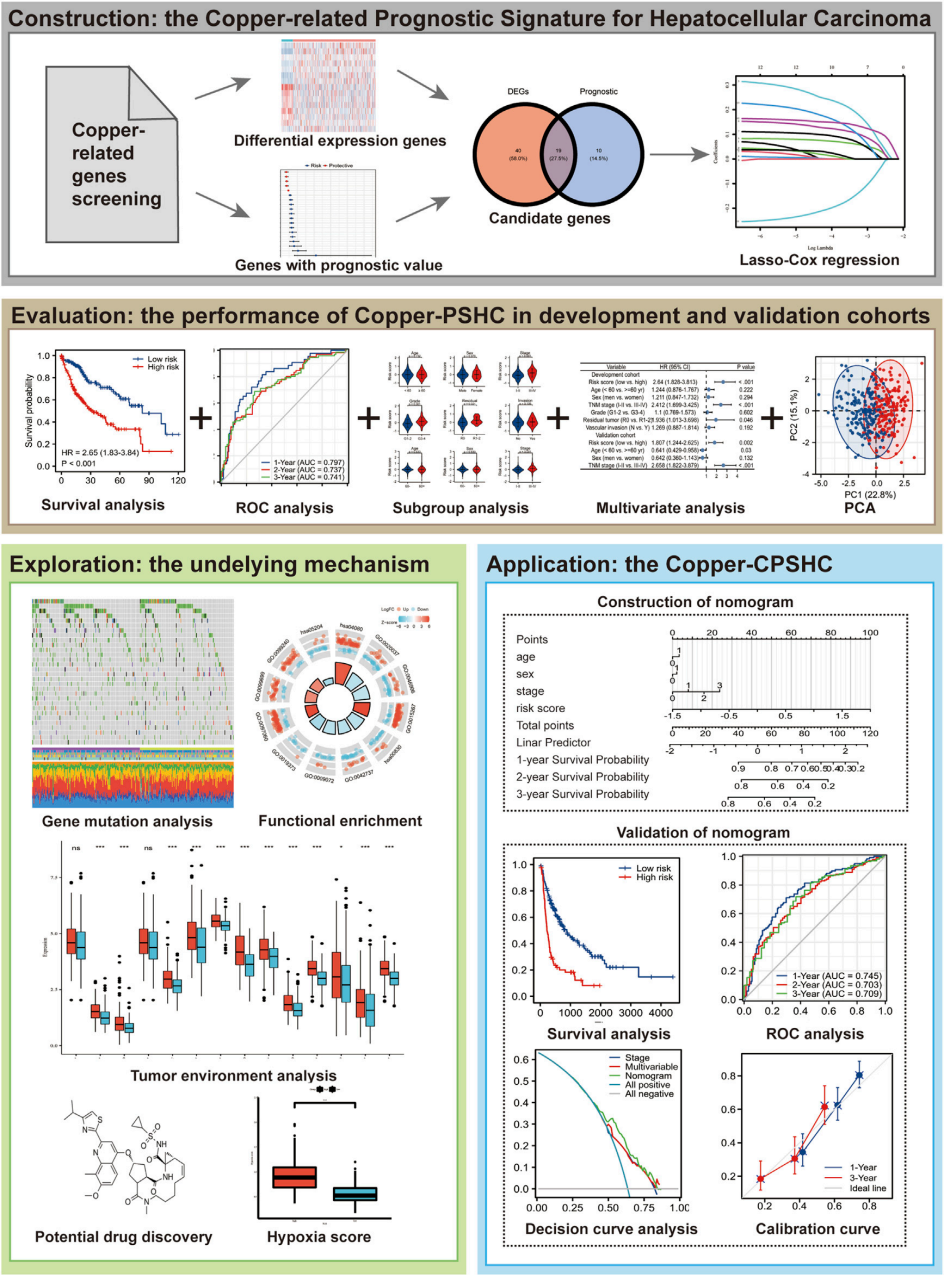
Flowchart of this study.

To construct a CRG-based prognostic signature (i.e., Copper-PSHC), we retrospectively analyzed the RNA sequencing data from two public HCC cohorts. The overall study design was depicted in Figure 1. HCC patients from The Cancer Genome Atlas (TCGA) liver and intrahepatic carcinoma dataset were utilized as the development cohort (Grossman et al., 2016) and those from Liver Cancer—RIKEN, JP (LIRI-JP) dataset were adopted as an independent validation cohort (The International Cancer Genome Consortium Data Portal, 2019). We excluded patients who had received systemic pharmaceutical therapy or radiotherapy prior to sample collection since such treatment may influence gene expression (Nakamura et al., 2013). Similarly, patients with multiple samples were also excluded to minimize the bias from tumor heterogeneity (Pe’er et al., 2021). Totally, we included 495 patients (351 men [70.9%], 304 aged ≥60 years [86.1%] and 355 at the cancer stage I/II [71.7%]), with 353 in the development cohort and 142 in the independent validation cohort (Supplementary Figure S1). Variables with less than 20% missing observations were imputed using multiple imputations by Chained Equations (White et al., 2011). Characteristics of participants following imputation were displayed in Supplementary Table S1. Details of case identification and imputation can be found in the Supplementary Method S1. The study was exempted from ethical review due to its use of de-identified, publicly available data.

### Construction of the Copper-PSHC and Copper-CPSHC

In accordance with the previous literature, ninety-six genes relevant to copper homeostasis, cuproptosis, and copper binding were screened (Supplementary Table S2) to construct the Copper-related Prognostic Signature (Copper-PSHC). First, the differentially expressed genes (DEGs) between tumor and adjacent non-tumor tissues with a false discovery rate (FDR) < 0.05 were identified in the development cohort. Then, univariable Cox analysis of overall survival (OS) was performed, with *p* < 0.05 chosen as the significance threshold, to determine the DEGs having prognostic value. Those prognostic DEGs measured in both cohorts were included to build the Copper-PSHC. Thereafter, we used the String Interaction Network to demonstrate the association between these genes (Szklarczyk et al., 2021) and estimated the correlation of gene expression. To minimize the risk of overfitting, a LASSO-Cox regression was applied to select the most contributing prognostic genes and to construct Copper-PSHC (Engebretsen and Bohlin, 2019). To calculate the risk score of each patient in the development and validation cohort, the normalized expression level of each gene and the corresponding regression coefficients generated from the development cohort were used. Then, patients were stratified into low-risk and high-risk groups based on the median risk score determined by the development cohort. Details of gene screening and Copper-PSHC construction can be found in Supplementary Method S2.

We further applied multivariable Cox regression, which integrated age, sex, cancer stage and Copper-PSHC risk score, to construct a composite prognostic model, Copper-CPSHC, in the development cohort. Age, sex, and cancer stage were treated as continuous variables. We also presented a nomogram of Copper-CPSHC to facilitate its use in clinical settings. The optimal cut-off value for classifying the patients into low- or high-risk groups was determined using a time-dependent ROC curve at 3 years of follow-up by Youden index (Fluss et al., 2005) in the development cohort. Details of Copper-CPSHC construction can be found in Supplementary Method S2.

### Validation of the Copper-PSHC and Copper-CPSHC

The primary endpoint was overall survival (OS), and the secondary endpoint was disease-free survival (DFS) which was not evaluated in the validation cohort owing to a lack of information on tumor recurrence. Proportional hazard assumption was not violated (Supplementary Table S3). The prognosis value of Copper-PSHC was first assessed as binary variables (high vs. low risk) in both cohorts by the univariable Cox proportional hazard model and represented with the Kaplan-Meier curve. Restricted mean survival time (RMST) was estimated for the high- and low-risk groups to quantify the life expectancy at 3 years of follow-up, while the difference between the two risk groups was determined by their disparity. Stratified analyses by age, sex, cancer stage were conducted for both cohorts, and the hazard ratio (HR) was merged using a fixed model. Then, we combined Copper-PSHC with age, sex and cancer stage in multivariable Cox proportional hazard regression to justify the prognostic value of Copper-PSHC. Adjusted HR (controlling for age, sex and cancer stage) was used to assess the performance of Copper-PSHC as a binary variable. We additionally performed time-dependent ROC analysis for OS and DFS to evaluate the predictive power of the model over time. The performance of Copper-PSHC continuous risk score was assessed by the area under the curve (AUC) of the time-dependent ROC curve at 3 years of follow-up. The concordance index (c-index) was also estimated to quantify the prognostic accuracy of the Copper-PSHC. Details of Copper-PSHC validation can be found in Supplementary Method S3.

Similarly, we performed univariable analysis for Copper-CPSHC using Kaplan-Meier curve and compared the RMSTs of two risk groups. The performance of Copper-CPSHC was also evaluated by HR (in binary scenario) and AUC of time-dependent ROC curve at 3 years of follow-up (in continuous form). The c-index was calculated. Additionally, calibration curves were depicted to characterize the discrimination of Copper-CPSHC. Decision curve analysis (DCA) was applied to measure the net benefits, which was compared with tumor stage and the clinical model. Details of Copper-CPSHC validation can be found in Supplementary Method S3.

### Annotation of Copper-PSHC

#### Somatic mutation analysis

In an attempt to explore the somatic mutations in high- and low-risk groups determined by Copper-PSHC in the development cohort, gene mutation data (available at cBioprotal, in “.maf” format) (Cerami et al., 2012) were analyzed. The 20 most commonly mutated genes were listed for each risk group and measured as frequency. Meanwhile, the total mutation frequency and tumor mutation burden (TMB) were also estimated. The waterfall plots were depicted to manifest the mutation landscape for the high- and low-risk groups by the “maftool” R package (Mayakonda et al., 2018).

#### Functional enrichment analysis

We performed the functional enrichment analysis in both the development and validation cohorts. Biological function and pathways regarding Copper-PSHC were analyzed based on Gene Ontology (GO) (The Gene Ontology Consortium, 2019) and the Kyoto Encyclopedia of Genes and Genomes (KEGG) database (Kanehisa et al., 2021) using the DEGs between high- and low-risk groups. Further, Gene Set Enrichment Analysis (GSEA) was conducted to determine the upregulated and downregulated cellular pathways in high-risk group compared with low-risk group, with an FDR <0.01 as the screening criteria (Kos et al., 2021). The function enrichment analysis was conducted by the “clusterProfiler” R package (Yu et al., 2012). We also estimated the HCC hypoxia score proposed by Hu et al. (2020) (Supplementary Method S4.1) for two risk groups.

#### Tumor microenvironment analysis

The correlation between Copper-PSHC and the tumor microenvironment (TME), which is comprised primarily of immune cells and stromal cells, was investigated in both the development and validation cohorts. We first applied ESTIMATE (Yoshihara et al., 2013) algorithm to depict the presence of immune cells, stromal cells, and tumor purity in two risk groups. Then, we adopted CIBERSORT (Newman et al., 2015), ssGESA (Barbie et al., 2009) and xCell (Aran et al., 2017) for the comparison of TME cells infiltration in two groups (Supplementary Method S4.2). Moreover, we also analyzed the expression of multiple cell markers related to immune checkpoint blockade (ICB) and exhausted T-cells (Kos et al., 2021) between high- and low-risk groups. These markers could represent the cell progressively losing function due to long-term exposure to persistent antigens or chronic inflammation (Wherry and Kurachi, 2015).

#### Exploration of potential therapy for HCC

We explored potential therapy for HCC patients in different risk groups via the CLUE (Subramanian et al., 2017) based on the DEGs between the high- and low-risk groups (Lamb et al., 2006). CLUE was developed based on the concept of CMap (connectivity map), where genes, drugs and disease states are connected. Hence, the potential drug to reverse the current disease status for the high-risk group can be identified by DEGs. See Supplementary Method S4.3 for detail. The potential drugs were selected with the criteria of enrichment score < −0.60 with *p* < 0.005 as the significance level (Yue et al., 2022).

#### Statistical analysis

All statistical analyses were carried out using R software (version 4.1.0). To compare the difference in proportions, Chi-square test was implemented. Student’s **t*-*** test was used for the comparison of continuous variables between two groups when the assumption of normal distribution was met; otherwise, its non-parametric counterpart Mann-Whitney *U* test was adopted. The correlation between gene expressions was examined by Spearman’s correlation coefficient. RMST was estimated by the “survRM2” package (survRM2, 2022) and c-index was calculated by the “survminer” package. A two-tailed *p* < 0.05 was deemed as statistically significant, unless otherwise specified.

## Results

### Construction of the Copper-PSHC

A total of 59 CRGs selected from 96 CRGs in previous literature were screened as DEGs between tumor and adjacent non-tumor tissues. By assessing the association of 59 DEGs with OS in the development cohort, 19 DEGS with prognostic value were determined as candidate genes (All *p* < 0.05, Supplementary Figures S2A–C). The correlation between these genes was shown in Supplementary Figure S2D and Supplementary Table S4, where *ALB* and *LOX* were identified as hub genes (Supplementary Figure S2E).

Thirteen DEGs out of the 19 candidate genes were measured in both cohorts and were included for further analysis. Finally, nine genes were selected by LASSO-Cox regression to construct the Copper-PSHC index, i.e., *CDKN2A*, *GPC1*, *LOX*, *MEMO1*, *SLC25A3*, *STEAP1*, *STEAP4*, *UBE2D2*, and *XIAP* (Supplementary Figure S3), where high expression of those genes portended a poor prognosis, with an exception for *STEAP4*. The associations of the nine genes with OS were presented in Supplementary Figure S4. According to the normalized expression level of each gene and the corresponding Cox regression coefficients, the Copper-PSHC risk score was generated for each individual as follows:

Copper-PSHC risk score = 0.069*expression level of *CDKN2A*+ 0.228*expression level of *GPC1* + 0.119*expression level of *LOX* + 0.089*expression level of *MEMO1* + 0.007*expression level of *SLC25A3* + 0.150* expression level of *STEAP1* −0.187*expression level of *STEAP4* + 0.147*expression level of *UBE2D2* + 0.017*expression level of *XIAP*.

**FIGURE 2:**
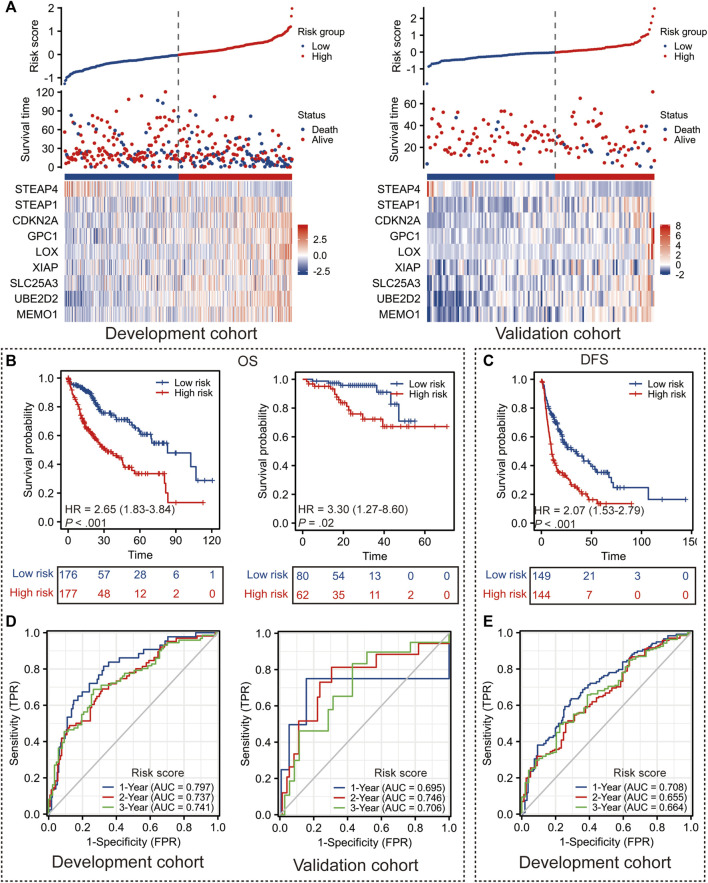
Performance of Copper-PSHC. **(A)** The distribution of Copper-PSHC risk score, survival time and the expression of each gene in Copper-PSCH in the development and validation cohort. **(B)** Kaplan-Meier survival curves showing the difference of overall survival (OS) and disease-free survival (DFS) between high- and low-risk groups in the development cohort. **(C)** Kaplan-Meier survival curves showing the difference of OS between high- and low-risk groups in the validation cohort. **(D)** Time-dependent ROC curves of 1-year, 2-year and 3-year OS and DFS for Copper-PSHC in the development cohort. **(E)** Time-dependent ROC curves and AUC in 1-year, 2-year and 3-year OS for Copper-PSHC in the validation cohort.

Taking the median risk score in the development cohort as the optimal cut-off value, the patients in the development cohort and the validation cohort were dichotomized into the low-risk (risk score < −0.021) and high-risk (risk score ≥ −0.021) groups (Figure 2A).

### Validation of the prognostic value of Copper-PSHC

Copper-PSHC demonstrated outstanding prognostic value. In terms of the primary endpoint, patients from the high-risk group demonstrated a significantly reduced OS (Figure 2B; HR: 2.65 [95% CI, 1.83–3.84] and 3.30 [95% CI, 1.27–8.60] in the development and validation cohorts, respectively) compared with those from the low-risk group. The 3-year RMSTs were significantly prolonged for the low-risk group in both the development (RMST difference: −7.4 [95% CI, −10.0 to −4.8] months) and validation cohorts (RMST difference: −4.1 [95% CI, −6.8 to −1.4] months) (Supplementary Table S5). After adjusting for age, sex and cancer stage, Copper-PSHC remained as an independent prognostic factor in the development cohort (HR: 2.33 [95% CI, 1.60–3.39]) as well as the validation cohort (HR: 3.11 [95% CI, 1.15–8.42]), as shown in Supplementary Table S6. Stratified analysis indicated that the Copper-PSHC maintained a prognostic factor for all subgroups, except for females (Supplementary Figure S5; Supplementary Table S7). Concerning the secondary endpoint, patients in the high-risk group had a significantly worse DFS than the low-risk group with (Figure 2C; HR: 2.07 [95%CI, 1.53–2.79]) or without adjusting for age, sex, vascular invasion and cancer stage (HR: 1.86 [95% CI, 1.36–2.52]) in the development cohort (Supplementary Table S8).

**FIGURE 3:**
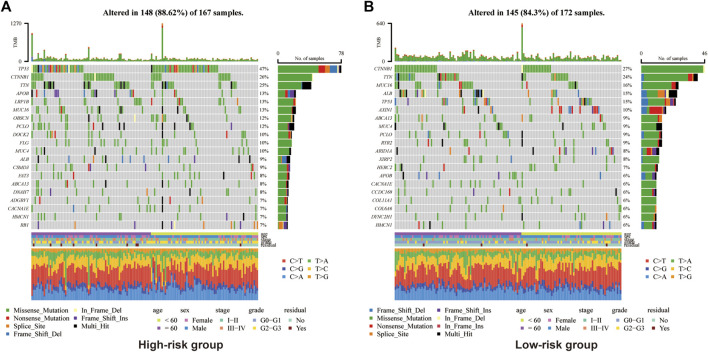
Somatic genes mutation analysis in the development cohort for high-risk group **(A)** and **(B)** low-risk group.

Time-dependent ROC curves for OS and DFS exhibited an excellent discriminative power of Copper-PSHC at 3 years of follow-up (Figure 2D). In development cohort, the AUC of OS achieved 0.80 [95% CI, 0.73–0.87], 0.74 [95% CI, 0.66–0.81] and 0.74 [95% CI, 0.67–0.82] at 1-, 2- and 3-year time points, respectively (Figure 3F). In validation cohort, the AUC value of OS remained 0.70 [95% CI, 0.30–1.10], 0.75 [95% CI, 0.60–0.89] and 0.71 [95% CI, 0.56–0.86] at 1-, 2- and 3-year time points, respectively (Figure 2D). For DFS, AUC yielded 0.71 [95% CI, 0.65–0.77], 0.66 [95% CI, 0.58–0.73] and 0.66 [95% CI, 0.58–0.75] at 1-, 2- and 3-year of follow-up, respectively (Figure 2E). Copper-PSHC also demonstrated accurate prediction for OS in both cohorts (c-index: 0.64 [95% CI, 0.60–0.68] and 0.68 [95% CI, 0.58–0.78] for development and validation cohorts, respectively; Supplementary Table S5).

A higher risk score was observed in patients with undesirable biological behaviors or processes, including more advanced TMN stage (III-IV, *p* < 0.001), margin residual (*p* = 0.021) and higher tumor grade (G3/G4, *p* < 0.001), as shown in Supplementary Figures S6A–I. Additionally, PCA analysis also divided patients into two directions, which was consistent with the classification pattern generated by Copper-PSHC (Supplementary Figures S6J, K).

### Construction and validation of the Copper-CPSHC

The Copper-CPSHC was derived after combining Copper-PSHC risk score with age, sex and TNM stage, leveraging the complementary value of molecular and clinical characteristics:

Copper-CPSHC risk score = [1.07292* Copper-PSHC risk score] + [0.12480* age] + [0.07879* sex] + [0.29818* stage].

Then, patients were classified into the high- (≥0.677) and low-risk (<0.677) groups according to the optimal cut-off determined by *Youden* index of the time-dependent ROC curve at 3-year follow-up in the development cohort.

The significant prolonged OS was observed among the low-risk group in the development cohort (HR: 4.27 [95% CI, 3.00–6.08]) and validation cohort (HR: 2.63 [95%CI, 1.09–6.32]) (Supplementary Figures S7A, C), with the difference of 3-year RMST of −11.8 (95% CI, −14.9 to −8.7) months and −4.0 (95% CI, −7.5 to −0.5) months for the development and validation cohorts, respectively (Supplementary Table S9). In development cohort, the AUC of the time-dependent ROC for OS reached 0.78 [95% CI, 0.72–0.84], 0.70 [95% CI, 0.63–0.78] and 0.73 [95% CI, 0.66–0.80] at 1-, 2- and 3-year time points, respectively (Supplementary Figure S7B). The AUC for OS in validation cohort yielded 0.72 [95% CI, 0.31–1.13], 0.75 [95% CI, 0.62–0.89] and 0.72 [95% CI, 0.56–0.87] at 1-, 2- and 3-year of follow-up, respectively (Supplementary Figure S7D). The c-index also demonstrated the validity of Copper-CPSHC on prognostic prediction in the development cohort (0.68 [95% CI, 0.63–0.72]) as well as the validation cohort (0.65 [95% CI, 0.53–0.77]), as shown in Supplementary Table S9.

We then constructed a nomogram to provide a handy quantitative instrument for clinical use (Supplementary Figure S7E). The calibration curves for 1-year and 3-year follow-up confirmed that the nomogram’s predicted probabilities were close to the observed probabilities (Supplementary Figures S7F, G), indicating the consistency between the prediction and the actual observation in both development and validation cohorts. Meanwhile, DCA demonstrated that the nomogram prediction possessed more area than the TNM stage and a clinical model including age, sex and cancer stage (Supplementary Figures S7H–K). Similar results were obtained for DFS in the development cohort (Supplementary Figure S8).

### Annotation of Copper-PSHC

#### Somatic mutation analysis

In view of the causal role of somatic mutation in cancer, we depicted the somatic mutation spectrum of the high- and low-risk groups determined by Copper-PSHC in the development cohort. In general, high-risk group was characterized by a higher mutation frequency (high-vs. low-risk: 88.6% vs. 84.3%, *p* < 0.01). The TMB was significantly higher for patients in the high-risk group (high-vs. low-risk:1.98 vs. 1.54, *p* < 0.05, Supplementary Figure S9). We exhibited the 20 most frequently mutated genes in two risk groups, respectively (Figure 3). The mutation related to undesirable biological behavior was enriched in high-risk group when compared to low-risk group, such as *TP53* (47% vs. 15%; OR: 5.15 [95% CI, 4.80–5.53]), a well-known carcinogenic gene of P53 pathway, and *DOCK2* (10% vs. 2%; OR: 4.76 [95% CI, 3.45–6.56]), an intercellular regulator of the Rho family GTPase, RAC1 (Sanui et al., 2003). The overview of mutations was also presented in Supplementary Figure S10 for both cohorts, revealing that missense mutation, SNP and C>T mutation were more common.

### Functional enrichment analysis

**FIGURE 4:**
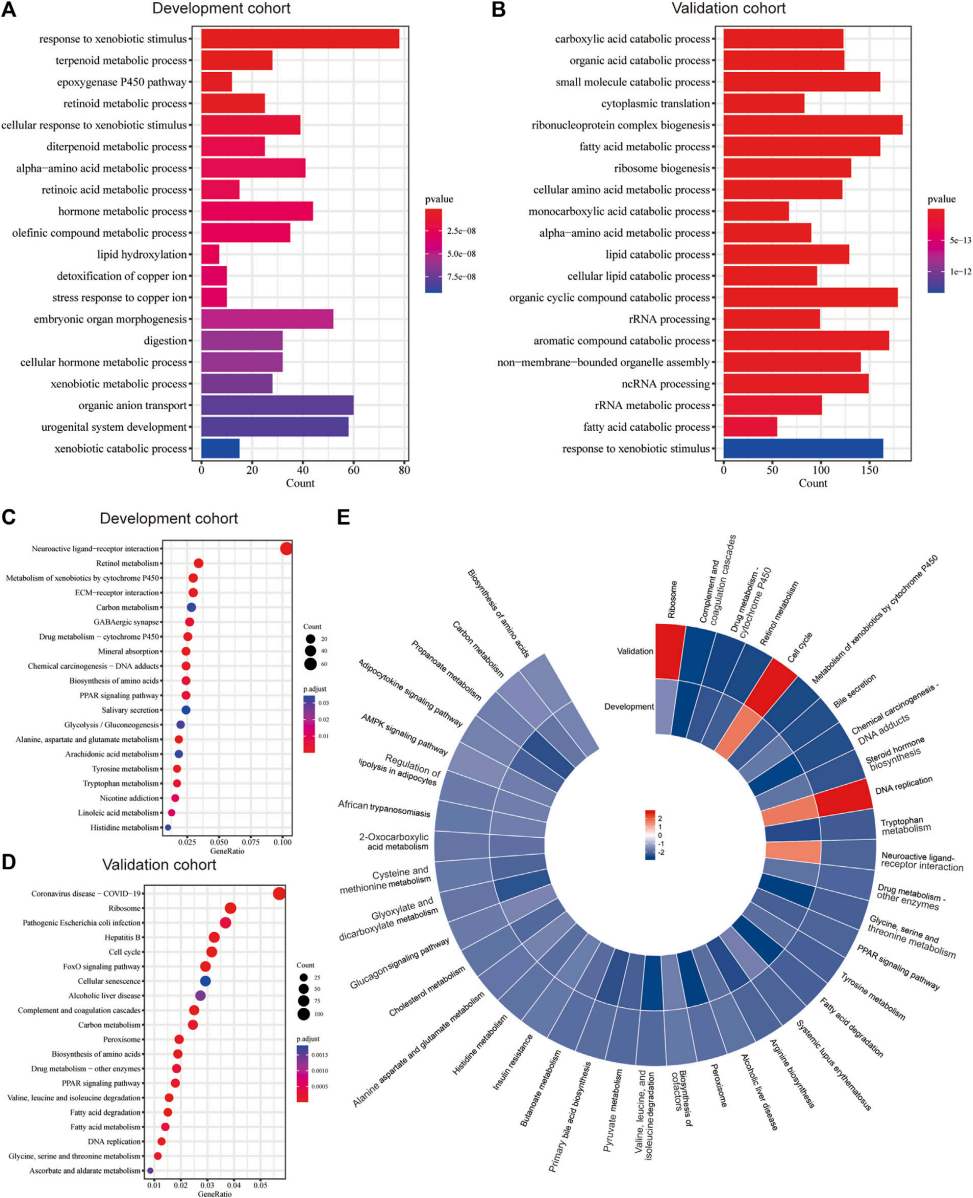
Functional enrichment analysis. The significant GO enrichment in the **(A)** development and **(B)** validation cohorts. The significant KEGG pathways in the **(C)** development and **(D)** validation cohorts. **(E)** The significantly upregulated and downregulated KEGG pathways in both cohorts according to GSEA.

The functional enrichment analysis highlighted the role of metabolic and biosynthesis pathways in the molecular mechanism regarding Copper-PSHC. GO enrichment revealed that the DEGs between high- and low-risk groups were related to metabolic processes, such as those regarding alpha-amino acid, hormone, and fatty acid (Figures 4A, B). Likewise, KEGG analysis demonstrated an enrichment of carbon metabolism and biosynthesis of amino acids in both cohorts (Figures 4C, D). Generally, most gene pathways were downregulated in the high-risk group when compared to the low-risk group, except for cell cycle and DNA replication pathways (Figure 4E). Considering that several pathways related to oxidation (e.g., pyruvate metabolism) were downregulated in the high-risk group, we additionally estimated the hypoxia score in two risk groups. The high-risk group was associated with a significantly increased hypoxia score than the low-risk group (1.39 vs. 1.08, *p* < 0.001; Supplementary Figure S11), suggesting a low oxygen status in HCC patients from the high-risk group.

### Tumor microenvironment analysis

**FIGURE 5:**
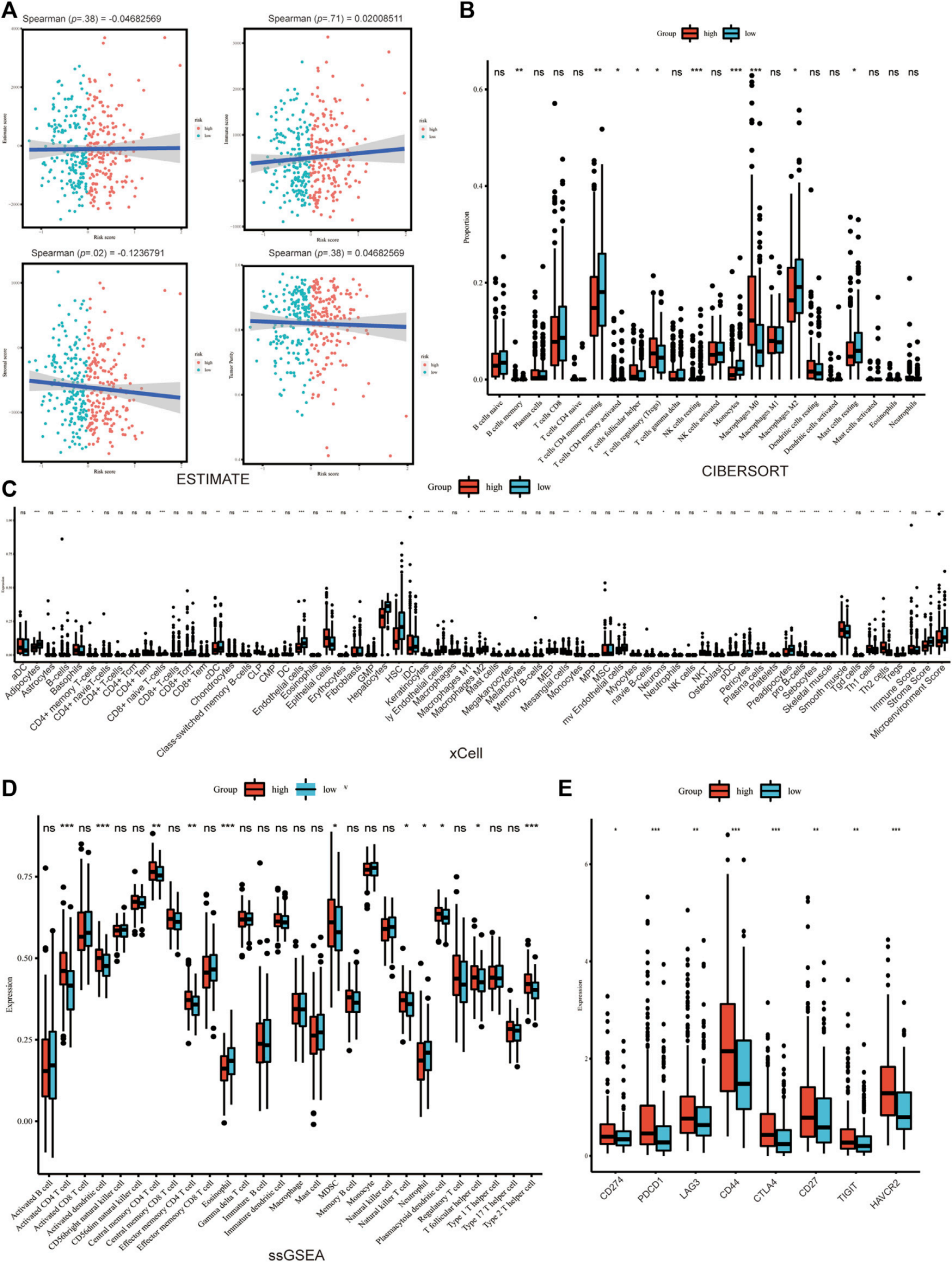
Tumor microenvironment analysis in development cohort. **(A)** The immune, stroma and Estimate score according to ESTIMATE algorithm. The differences of TME cells infiltration between high- and low-risk groups according to **(B)** Cibersort, **(C)** xCELL and **(D)** ssGSEA. **(E)** The significant difference of the expression of the cell marker related to ICB between groups. (where *p < 0.05, **p < 0.01, ***p < 0.001).

The association between Copper-PSHC and tumor microenvironment was shown in Figure 5 and Supplementary Figures S12, S13. Overall, a significantly negative correlation was demonstrated between stroma score and Copper-PSHC risk score in both development and validation cohorts, with Spearman correlation coefficient estimated as −0.12 (*p* = 0.02) and −0.56 (*p* < 0.001), respectively. That echoed the observation of high stroma cell infiltration in the low-risk group (both *p* < 0.001). The enrichment of stroma cells in low-risk group is primarily driven by (pre-) adipocytes, pericytes, lymphatic endothelial cells, and skeletal muscle cells (Figure 5C; Supplementary Figure S12C).

The immune score was neither significantly correlated with Copper-PSHC risk score nor differed between the two risk groups. However, between-group differences were observed for certain immune cell infiltration, despite disagreement using different algorithms. For example, CD4+T cells memory resting and conventional dendritic cell (cDC) were highly infiltrated among the low-risk group, while CD4+T cells memory activated, natural killer T cell (NKT), type 2T helper cell (Th2) were enriched among the high-risk group (All *p* < 0.05; Figures 5B–D; Supplementary Figures S12B–D).

Additionally, the expression of markers related to ICB and T cell exhaustion was significantly elevated for the high-risk group in the development cohort, including CD274, PDCD1, LAG3, CD44, CTLA4, CD27, TIGHT and HAVCR2 (All *p* < 0.05; Figure 5E; Supplementary Figure S13A, B). This result hinted that immunotherapy might benefit the high-risk group.

### Identification of potential drugs

Potential treatments were explored via CLUE based on the DEGs between high- and low-risk groups. Under our screening criteria, flavokavain-b, simeprevir, BRD-K88741031, RAF-265 butein and ASC-J9 were discovered as potential drugs for the high-risk group (Table 1). EGFR inhibitor was the primary mode of action for the drugs above. Also, carcinogens, HCV inhibitors, tyrosine kinase inhibitors, RAF inhibitors, Src inhibitors and androgen receptor agonists were the potential targets for treating the high-risk group as well.

**TABLE 1:** Potential small molecules drug for high-risk HCC treatment.

CMap name	MOA	Target	Enrichment^a^
Flavokavain-b	Carcinogen	IKBKB	−0.6015
Simeprevir	HCV inhibitor	CYP2C19, CYP2C8, SLCO1B3, CYP1A2, CYP3A4	−0.6017
BRD-K88741031	Tyrosine kinase inhibitor, EGFR inhibitor	EGFR	−0.6018
RAF-265	RAF inhibitor, VEGFR inhibitor	BRAF, KDR, KIT, PDGFRB, RAF1	−0.6116
Butein	EGFR inhibitor, Src inhibitor	ACE, CXCL8, IL6, SIRT1, SRD5A1, SRD5A2, TNF	−0.6118
ASC-J9	Androgen receptor agonist	AR	−0.6487

Abbreviations: HCC, hepatocellular carcinoma; CMap, Connectivity map.

^a^ The enrichment score represents the similarity between drugs and current biological process or disease status. A negative score indicates that the drug could reverse the disease status and have potential therapeutic value.

## Discussion

In this study, we developed and validated a 9-CRG prognostic signature, Copper-PSHC, for HCC patients. We also combined clinical features including age, sex and cancer stage with Copper-PSHC to build a composite prediction model, Copper-CPSHC, for clinical prognostic stratification. Both Copper-PSHC and Copper-CPSHC were demonstrated as reliable tools with excellent prognostic value in the development and validation cohorts. Beyond that, extensive work has been carried out for the annotation of Copper-PSHC.

An increasing number of prognostic models for HCC have been proposed. For example, Liang et al. (2020) developed a ferroptosis-related gene signature for OS prediction. Tang et al. (2022) constructed an immunological phenotype-related gene signature for predicting prognosis. Xu et al. (2021) fitted a ferroptosis-related nine-lncRNA signature for predicting prognosis and immune response. Compared to those models, Copper-PSHC had an exceptional advantage in predicting OS and DFS in both the development and validation cohorts. We also provided a nomogram combining clinical variables and risk score for ready clinical application. Additional advantage of our study includes examining the potential of immunotherapy in the management of HCC. On balance, we developed a reliable copper-related model to predict prognosis which is of high significance in clinical decision-making.

Our study confirmed previous findings on the association between 9 genes in Copper-PSHC with cancer. It is collectively speculated that these genes could play crucial roles in tumor development and/or progression, and therefore own considerable prognostic value for HCC. Previous research on network-based prioritization of HCC markers by module detection and ranking has demonstrated the diagnostic value of CDKN2A (Shang and Liu, 2021). Besides, Luo et al. (2021) found that *CDKN2A* was highly expressed in HCC and associated with a decreased OS via facilitating the proliferation of cancer cells and inhibiting apoptosis. *LOX* was the mediator of remodeling of the extracellular matrix cross-linking, thereby contributing to the angiogenesis (Sun et al., 2022). *XIAP* could induce the resistance to apoptosis, providing survival advantage to the metastatic tumor cells (Shi et al., 2008). As a cell surface heparan sulfate proteoglycan, *GPC1* was found to exhibit a mitogenic response to multiple heparin-binding growth factors and lead to progression in breast cancer (Matsuda et al., 2001). *GPC1* was also used as a potent predictive biomarker for the general prognosis of HCC (Wang JY. et al., 2021). Analogously, *SLC25* protein family, *MEMO* and *STEAP* were identified as potential biomarkers for prognosis (Gomes et al., 2012; MacDonald et al., 2014; Rochette et al., 2020). However, much uncertainty still exists about the opposite roles of *STEAP1* and *STEAP4* in HCC.

To our knowledge, Copper-PSHC was the first prognostic prediction model related to copper binding, copper homeostasis and cuproptosis. Recently, the association between cuproptosis and HCC has been elucidated. As a mineral nutrient, the significance of copper for various physiological processes has been well recognized across the animal kingdom to human (Ge et al., 2022). Copper functions as a crucial cofactor for enzymes that mediate a range of cellular activities including mitochondrial respiration and antioxidant defense (Luo et al., 2021); therefore, copper homeostasis was critical for cellular growth and maintenance. Of note, increasing evidence demonstrated that copper and the disruption of copper homeostasis were involved in oncogenesis (Brady et al., 2014; Shanbhag et al., 2019; Llovet et al., 2021). This supports the argument that copper may activate several proangiogenic factors such as vascular endothelial growth factor, fibroblast growth factor 2, tumor necrosis factor and interleukin-1 (Gérard et al., 2010; Das et al., 2022; Ge et al., 2022). Meanwhile, emerging cancer therapeutics targeting copper and copper-dependent signaling pathways exhibit significant promise, including copper chelators to inhibit cuproplasia and copper supplementation to promote cuproptosis. Taken together, it is of great importance to assess the copper status and characterize the landscape of copper in HCC patients. Regarding this, our study might provide valuable insights for understanding HCC and the management of HCC patients through the lens of copper homeostasis.

Our findings of correlation between high expression of CDKN2A and poor prognosis in HCC further evidenced the antitumor effect of cuproptosis, a distinct form of cell death dependent on intracellular copper accumulation, where FDX1 and protein lipoylation serve as the hub regulators and CDKN2A serve as a negative regulator (Tsvetkov et al., 2022). Besides, we found that pyruvate metabolism related to the TCA cycle, which is a necessary condition for copper-induced cell death, was downregulated in the high-risk group. This finding again echoes the currently available evidence that cuproptosis may contribute to the inhibition of tumor growth and recurrence and thus a favorable prognosis by killing cancer cells modulated by the tumor microenvironment. It was reported that copper-dependent cell death was attenuated under the hypoxic condition, leading to an increased risk of tumor growth and progression. In support of this, we analyzed the hypoxic level in high- and low-risk groups and found that tumor cells in high-risk group were prone to be in hypoxic environments. Our results reveal that evaluation of the hypoxia level may help guide HCC treatment, especially the copper-targeted therapeutic strategies. Patients at high-risk are warranted for more intensive or personalized treatment strategies. For example, regorafenib and cabozantinib should be additionally used as systemic therapy for those high-risk patients (Solimando et al., 2022). Also, more targeted clinical studies need to be conducted in the high-risk populations we identified. Elesclomol, in combination with paclitaxel for melanoma, was a good example where statistically significant improvement was observed for patients with normal baseline levels of lactate dehydrogenase (LDX) (O'Day et al., 2013).

Additionally, we found that the high-risk group held a higher mutation frequency and TMB. *TP53*, as the most common mutation in the high-risk group, has been reported to be associated with undesirable biological behaviors including high AFP, advanced tumor stage, vascular invasion, poor tumor differentiation, and poor Child-Pugh class (Long et al., 2019). Other undesirable mutations were also enriched in the high-risk group, such as DOCK2 which regulates Rac activation and cytoskeletal reorganization (survRM2, 2022), accounting partly for poor prognosis in the high-risk group as well.

Functional enrichment analysis suggested that the metabolic and biosynthetic processes were instrumental in Copper-PSHC. The downregulated pathways related to amino acid and lipid metabolism implied that the high-risk group featured low metabolic activity. According to the metabolism-associated molecular classification of HCC, high metabolic activity was related to α‐fetoprotein (AFP) expression and good prognosis (Yang et al., 2020). Another model proposed by Désert et al. classified HCC as “ECM‐type,” “STEM‐type,” “PV‐type” and “PP‐type.” Among them, “PP-type” characterized by high lipid and bile salt metabolism has displayed low proliferation and favorable prognosis (Ng et al., 2017). Given that liver is the primary handler of amino acid and lipid metabolism, we supposed that maintenance of high metabolic activity in the low-risk group conferred high chances of preserving normal liver phenotype, which may lead to less aggressive clinical and biological behaviors (Phillips, 2022).

Importantly, we explored the association between Copper-PSHC and tumor microenvironment. Overall, more infiltration of stromal cells was found in the low-risk group, suggesting their low degree of tumor purity and well differentiation. Although no significant difference in overall immune cell infiltration was observed between high- and low-risk groups, great disparities exist for certain types of immune cells. Patients in the high-risk group were prone to possess more activated cells and T helper cells. Generally, the infiltration of immune cells including T cells, macrophages, and B cells would conduct to the desirable prognosis (Galluzzi et al., 2020), especially in colorectal cancer (Picard et al., 2020) and breast cancer (Wang S. et al., 2021). Such inconsistency led us to further explore the expression of receptors in immune cells. As a result, high expression of immune regulators including CTLA-4 and PD-L1 was found in the high-risk group, which suggested that the infiltrated T cells in the high-risk group were mainly exhausted T cells. T cell exhaustion characterized by a loss of effector functions and memory T cell properties would hamper optimal control of tumors (Blank et al., 2019) and thereby account for poor prognosis of the high-risk group. Notably, the enrichment of cell markers related to ICB lent credence to the availability of immunotherapy for the high-risk subpopulation. This implication was also supported by the finding that higher TMB was shown in the high-risk group. Tumors with high TMB were more likely to respond to ICB agents (Chan et al., 2019) because greater tumor load could enhance the likelihood of being recognized by T cells (Litchfield et al., 2021).

Apart from the advantages and implications of this study as discussed above, several limitations also require due consideration. Firstly, restricted to the retrospective study, we may introduce the selection bias such as the exclusion of some patients who lacked data or were ineligible for sequencing, particularly those patients who are unable to undertake surgery treatment due to comorbidities or tumor metastasis. Secondly, the sample size was not considered large enough owing to the limited number of HCC patients in advanced stage. Further optimization of the Copper-PSHC index taking the stages of patient population into account is warranted for future studies. Thirdly, due to the unavailability of other clinical data including treatment history, comorbidities and laboratory values, and radiomic data, we were unable to incorporate more variables into our composite model. Notwithstanding, the index Copper-CPSHC developed in this study already owns satisfactory prognostic prediction value. Fourthly, with an aim of ensuring the robustness of results in tumor microenvironmental analysis, most immune cells were analyzed by two or more algorithms. However, certain cells, stromal cells for instance, were exclusively analyzed by xCell. Fifthly, the screening of potential drugs in this study is explorative and more efforts are warranted to further validate these finding in future studies. Lastly, the validation was performed using the public cohorts and we only provided a brief discussion of the potential mechanism of the genes in the model. More prospective studies and further exploration of the biological mechanism in the context of copper and HCC was in warranted.

In conclusion, this study constructed a copper-related signature, Copper-PSHC, based on nine CRGs, which has been subsequently demonstrated to be a reliable biomarker for prognostic prediction. We then move forward to examine the hypothesis of metabolic process and tumor immunity being the mechanisms of this signature, illuminating the potential of certain small molecular drugs and immunotherapy for better management of HCC patients. It is envisaged that further investigation using different research tools will help to elucidate the underlying mechanism and verify its clinical utility in the real world.

## Data Availability

The original contributions presented in the study are included in the article/Supplementary Material, further inquiries can be directed to the corresponding authors.
