# Supplementary material for: Development and validation of a copper-related gene prognostic signature in hepatocellular carcinoma
Source: Front Cell Dev Biol. 2023 Jul 18;11:1157841. doi: 10.3389/fcell.2023.1157841 (PMC10393034; doi:10.3389/fcell.2023.1157841)

SUPPLEMENTARY MATERIALS

Development and Validation of a Copper-related Gene Prognostic Signature in Hepatocellular Carcinoma

Haoting Shi[1,†], Jingxuan Huang[2,†], Xue Wang[3,†], Runchuan Li[2], Yiqing Shen[4], Bowen Jiang[5], Jinjun Ran[6], Rong Cai[1], Fang Guo[7,*], Yufei Wang[2,*], Gang Ren[8,*]

[1] Department of Radiation Therapy, Ruijin Hospital, Shanghai Jiao Tong University School of Medicine, Shanghai, China

[2] Department of Clinical Medicine, Shanghai Jiao Tong University School of Medicine, Shanghai, China

[3] Department of Pathology, Ruijin Hospital, Shanghai Jiao Tong University School of Medicine, Shanghai, China

[4] Department of Computer Science, Johns Hopkins University, Maryland, USA

[5] College of Biophotonics, South China Normal University, Guangzhou, China

[6] School of Public Health, Shanghai Jiao Tong University School of Medicine, Shanghai, China

[7] School of Public Health, Li Ka Shing Faculty of Medicine, The University of Hong Kong, Hong Kong, China

[8] Department of Radiology, Xinhua Hospital, Shanghai Jiao Tong University School of Medicine, Shanghai, China

[†] Haoting Shi, Jingxuan Huang, and Xue Wang contributed equally to this article.

* **Corresponding Author:** Gang Ren, PhD, MD, Department of Radiology, Xinhua Hospital, Shanghai Jiao Tong University School of Medicine, Shanghai, China, Shanghai, 200092, China (rengang@xinhuamed.com.cn); Yufei Wang, Master, Division of Cardiology, State Key Laboratory for Oncogenes and Related Genes, Renji Hospital, School of Medicine, Shanghai Jiao Tong University, Shanghai Cancer Institute, Shanghai, 200127, China (yufei8828@gmail.com); Fang Guo, PhD, School of Public Health, Li Ka Shing Faculty of Medicine, The University of Hong Kong, Pok Fu Lam, Hong Kong SAR, China (guof0818@hku.hk)

Supplementary Methods

Supplementary method 1. Case identification and imputation

Hepatocellular carcinoma (HCC) was ascertained by *the International Classification of Diseases for Oncology*, Third Edition using site code C22.0 and histology code 8170/3-8175/3. We excluded Intrahepatic cholangiocarcinoma (8160/3), combined hepatocellular-cholangiocarcinoma (8180/3), and papillary transitional cell carcinoma (8130/3) due to the distinct prognosis and biological behavior with HCC.

To make full use of all available data with missing observations in the development cohort, we applied imputation by Chained Equations [1] under the assumption of "missing at random". We included Copper-PSHC risk score (as a continuous variable), age, sex, tumor grade, margin residual, vascular invasion, and stage in the imputation model, with a random seed of 5,020,027. The missing observations were 1.4%, 7.4%, 15.3%, and 6.8% for tumor grade, margin residual, vascular invasion, and stage, respectively. Only variables with less than 20% missing observations were imputed.

Supplementary method 2. Model construction

2.1 Differentially expressed genes (DEGs) selection

For the initial screening of copper-related genes, we searched PubMed for studies with the keywords of (“copper homeostasis” OR “copper binding” OR “cuproptosis”) included in the title or abstract. Then, two researchers (HS and JH) read the title and abstract to determine whether the articles fit the purpose of our study, respectively. When there was a disagreement, a third researcher (XW) would judge it. Finally, ten articles that were most consistent with our study were included. We enumerated the copper-related genes mentioned in these articles and excluded duplicates. Genes related to copper included in the study and the corresponding references were presented in Supplementary Table 2.

The DEGs between tumor tissues and non-tumor tissues were identified by “DESeq2” [2] R package in the development cohort firstly, with the fold change > 1 and a false discovery rate < 0.05. To minimize the false discovery rate of the DEGs, we then used “limma” R package [3] to identify the DEGs between the two types of tissues. The intersection of the DEGs identified by “DESeq2” and “limma” and measured in both cohorts was treated as the DEGs for downstream analyses.

2.2 Development of Copper-PSHC

We used the least absolute shrinkage and selection operator (LASSO) Cox regression to select the most contributing prognostic genes among the rest of the 13 genes to establish Copper-PSHC for prediction of overall survival. The LASSO is a valuable method for selecting the predictors with the most contributing values and fitting a generalized linear model based on L1 regularization. [4] It is widely used in regression analysis with high-dimensional inputs to prevent overfitting. LASSO has been applied to the Cox regression model for survival analysis. [5] The penalty parameter, λ, was determined by 10-fold cross-validation following the minimum criteria, i.e., the optimal λ is where the value of the smallest partial likelihood deviance was observed. Copper-PSHC was then constructed as follows:

$$\text{Copper-PSHC Risk score} = \sum_{i=1}^{n} \beta_i * \text{Normalized expression level of gene}_i,$$

where β is the corresponding Cox regression coefficient and the gene expression level was normalized using the Z-score method for each cohort.

Finally, nine genes were included in the Cox regression model. Our model did not violate the 10EPV principle [6] as the observed number of events (death) was 129 and 119 in the development and validation cohort. The analysis was performed with the “glmnet” R package [7].

2.3 Development of Copper-CPSHC

To develop a personalized prognostic model for HCC patients, we combined clinical factors, including age, sex, and stage with Copper-PSHC to construct a composite prognostic model, Copper-CPSHC, using multivariate cox regression in the development cohort. Age, sex, and stage were treated as continuous variables. Age < 60 years were coded as 0 and ≥ 60 years were coded as

1. Male were coded as 0 and female were coded as 1. Stage was coded as I (0), II (1), III (2), and IV (3). The Copper-CPSHC was calculated as follows:

Copper-CPSHC Risk score = 1.07292 * Copper-PSHC risk score + 0.12480 * Age + 0.07879 * Sex + 0.29818 * Stage

Supplementary method 3. Model validation

3.1 Validation of Copper-PSHC

The risk score generated by Copper-PSHC was estimated for each individual in both cohorts. Then, we adopt the median score in the development cohort as a cut-off value to classify HCC patients into the high- (≥ median) and low-risk (< median) groups.

We conducted univariate analyses to evaluate the prognostic value of Copper-PSHC in both cohorts. Then, stratified analyses by age (< 60 vs. ≥ 60 years), sex (male vs. female), and stage (I-II vs III-IV) were then performed for both cohorts, and the hazard ratio (HR) was merged. Considering the small sample size of stage IV patients in both cohorts (1.4% and 7.0 %for the development and validation cohort, respectively), we combined stage III-IV patients as one group, while stage I-II was another.

The proportional hazards assumption was tested with Grambsch and Therneau method [8-9]. In the overall population of the two cohorts, the proportional hazards assumption was not violated (Supplementary Table S3). However, considering the statistical examination was not robust in subgroups due to the small population, where the violation of proportional hazards assumption may exist, we reported HR estimated either by cox model or log-rank test (annotated in Supplementary Figure 5). Further, the HR estimated with the log-rank test was merged by SAS (SAS Institute, Cary, NC) using a fixed model.

Further, we included age (< 60 vs. ≥ 60 years), sex (male vs. female), and stage (I-II vs. III-IV) in multivariate analyses to justify whether Copper-PSHC was independently associated with survival outcomes.

Time-dependent ROC analyses for time-to-event outcomes, i.e., OS and DFS, were performed to test the predictive power of Copper-PSHC over time. Time-dependent ROC analysis was used to characterize the predictive accuracy of a predictor when the outcome was censored data [10]. Considering the median follow-up time in both cohorts was less than 3 years (28.0 months [IQR 23.9 to 33.8 months] and 28.5 months [IQR 24.6 to 30.5 months] for the development and validation cohort, respectively), we set 1, 2 and 3 years as time point to evaluate the predictive power by estimating the area under the curve.

Additionally, restricted mean survival times (RMSTs) were estimated for high- and low-risk groups determined by Copper-PSHC to quantify the long-term survival benefit [11-12]. We reported the overall RMSTs and the 36-month RMSTs. The concordance index (c-index) was estimated to quantify the prognostic accuracy. RMST was estimated by the "survRM2" package. [13] C-index was calculated by the R "survminer" package. [14]

3.2 Validation of Copper-CPSHC

The risk score generated by Copper-CPSHC was estimated for each individual in both cohorts. Then, the optimal cut-off value classifying the high- and low-risk group was determined according to the Youden index of the time-dependent ROC curve at 3 years.

Similar to the validation of Copper-PSHC, we performed univariate analysis and represented in Kaplan-Meier curves. Time-dependent ROC analyses were also performed at 1, 2, and 3 years. RMSTs and C-index were also estimated. We additionally evaluated the model calibration with calibration curves [15], which compared the predicted risk with the observed risk. Also, we conducted a decision curve analysis (DCA). [15-16] We compare the net benefits of Copper-CPSHC with TNM stage and a multivariate model including age, sex, and stage.

Supplementary method 4. Annotation of Copper-PSHC

4.1 Hypoxia analysis

The HCC hypoxia score of the high- and low-risk groups were calculated as bellow [17]:

> Hypoxia score = 0.0376* expression of *HAVCR1* + 0.0337* expression of *PSRC1* + 0.1417* expression of *CCNJL* + 0.0530* expression of *PDSS1* + 0.0316* expression of *MEX3A* + 0.2148* expression of *EID3* + 0.0148 * expression of *EPO* + 0.0081* expression of *PLOD2* + 0.0296* expression of *KPNA2* + 0.0381* expression of *CDCA8* + 0.2877* expression of *ADAMTS5* + 0.0187* expression of *SLC1A7* + 0.0065* expression of *PIGZ*

A higher hypoxia score indicates a lower oxygen status, which associated with a poorer prognosis [18-19]. Previous studies found that hypoxia promotes proliferation, metastasis, angiogenesis, resistance to radiotherapy and chemotherapy of HCC [20-21]. Of note, the assessment of hypoxia was limited in the development cohort because some of the above genes were not measured in the validation cohort.

4.2 Tumor microenvironment analysis

Four algorithms, ESTIMATE [22], CIBERSORT [23], ssGSEA [24] and xCell [25], were applied in both the development and validation cohort to analysis the tumor microenvironment.

ESTIMATE is a gene-signature-based algorithm to infer the fraction of stromal and immune cells. Stromal score and immune score were estimated to quantify the presence of stromal and immune cell, respectively. Then these two scores were combined to generate an ESTIMATE score, which representing the tumor purity. CIBERSORT is a deconvolution-based approach to infer the proportions of 22 immune cells based on linear support vector regression. ssGSEA is an approach which converts gene expression profiles of individual samples into gene set enrichment profiles to calculate immune cell infiltration scores. Similarly, xCell is a marker-gene-based approach to infer 64 immune and stromal cell types. This approach was based on reliable gene signature (marker) and ssGSEA algorithm. Also, an immune, stroma and microenvironment score were generated by xCell to describe the infiltration of immune and stroma cells, and to measure the abundance of microenvironment.

In our analysis, significant differences (for proportion and expression) were only claimed if they were found in both the development and validation cohorts. For those cells estimated by more than one algorithm, the findings were considered robust only when all algorithms reach consensus.

4.3 Exploration of potential therapy for HCC

The online website CLUE (available at: https://clue.io/) was used to query the potential drugs. CLUE was based on the concept of CMap (Connectivity map), whereby genes, drugs and disease states are connected by virtue of common gene-expression signatures [26-27]. Hence, the potential therapy to reverse the disease status for high-risk group can be predicted by DEGs.

The upregulated and downregulated gene symbol between high- and low-risk were upload to the website. The parameters of query were select as “Gene expression (L1000), touchstone”. Then, an enrichment score ranging from -1 to 1 will be generated, representing the similarity between drugs and current biological process or disease status. A positive score indicates that drugs could induce the biological phenomena, while a negative score represents that the drug could reverse the disease status and have potential therapeutic value. The potential drugs were selected with the criteria of $P < .05$ and enrichment score < -0.60.

Supplementary References

1. White IR, Royston P, Wood AM. Multiple imputation using chained equations: Issues and guidance for practice. Stat Med. 2011 Feb 20;30(4):377-99.
2. Love MI, Huber W, Anders S. Moderated estimation of fold change and dispersion for RNA-seq data with DESeq2. Genome Biol. 2014;15(12):550.
3. Ritchie ME, Phipson B, Wu D, Hu Y, Law CW, Shi W, Smyth GK. limma powers differential expression analyses for RNA-sequencing and microarray studies. Nucleic Acids Res. 2015 Apr 20;43(7):e47.
4. Goeman JJ. L1 penalized estimation in the Cox proportional hazards model. Biom J. 2010 Feb;52(1):70-84.
5. Tibshirani R. The lasso method for variable selection in the Cox model. Stat Med. 1997 Feb 28;16(4):385-95.
6. Peduzzi P, Concato J, Kemper E, Holford TR, Feinstein AR. A simulation study of the number of events per variable in logistic regression analysis. J Clin Epidemiol. 1996 Dec;49(12):1373-9.
7. Sun C, Zhu B, Zhu S, Zhang L, Du X, Tan X. Risk Factors Analysis of Bone Mineral Density Based on Lasso and Quantile Regression in America during 2015-2018. Int J Environ Res Public Health. 2021 Dec 30;19(1):355.
8. Ledermann JA, Embleton-Thirsk AC, Perren TJ, Jayson GC, Rustin GJS, Kaye SB, Hirte H, Oza A, Vaughan M, Friedlander M, González-Martín A, Deane E, Popoola B, Farrelly L, Swart AM, Kaplan RS, Parmar MKB; ICON6 collaborators. Cediranib in addition to chemotherapy for women with relapsed platinum-sensitive ovarian cancer (ICON6): overall survival results of a phase III randomised trial. ESMO Open. 2021 Apr;6(2):100043
9. Stensrud MJ, Hernán MA. Why Test for Proportional Hazards? JAMA. 2020 Apr 14;323(14):1401-1402.
10. Heagerty PJ, Lumley T, Pepe MS. Time-dependent ROC curves for censored survival data and a diagnostic marker. Biometrics. 2000 Jun;56(2):337-44.
11. Zhang C, Wu Y, Yin G. Restricted mean survival time for interval-censored data. Stat Med. 2020 Nov 20;39(26):3879-3895.
12. Parikh RB, Min EJ, Wileyto EP, Riaz F, Gross CP, Cohen RB, Hubbard RA, Long Q, Mamtani R. Uptake and Survival Outcomes Following Immune Checkpoint Inhibitor Therapy Among Trial-Ineligible Patients With Advanced Solid Cancers. JAMA Oncol. 2021 Dec 1;7(12):1843-1850.
13. survRM2: Comparing restricted mean survival time. R package version 1.0-4. https://CRAN.R-project.org/package=survRM2.
14. survminer: Drawing Survival Curves using 'ggplot2'. R package version 0.4.9. https://cran.r-project.org/web/packages/survminer/index.html.
15. Vickers AJ, Van Calster B, Steyerberg E. Decision Curves, Calibration, and Subgroups. J Clin Oncol. 2017 Feb;35(4):472-473.
16. Vickers AJ, Elkin EB. Decision curve analysis: a novel method for evaluating prediction models. Med Decis Making. 2006 Nov-Dec;26(6):565-74.

17. Hu B, Yang XB, Sang XT. Development and Verification of the Hypoxia-Related and Immune-Associated Prognosis Signature for Hepatocellular Carcinoma. J Hepatocell Carcinoma. 2020 Nov 11;7:315-330.
18. Jing X, Yang F, Shao C, Wei K, Xie M, Shen H, Shu Y. Role of hypoxia in cancer therapy by regulating the tumor microenvironment. Mol Cancer. 2019 Nov 11;18(1):157.
19. Rankin EB, Giaccia AJ. Hypoxic control of metastasis. Science. 2016 Apr 8;352(6282):175-80.
20. Wu XZ, Xie GR, Chen D. Hypoxia and hepatocellular carcinoma: the therapeutic target for hepatocellular carcinoma. J Gastroenterol Hepatol. 2007;22(8):1178–1182.
21. Xia S, Pan Y, Liang Y, Xu J, Cai X. The microenvironmental and metabolic aspects of sorafenib resistance in hepatocellular carcinoma. EBioMedicine. 2020 Jan;51:102610.
22. Yoshihara K, Shahmoradgoli M, Martínez E, Vegesna R, Kim H, Torres-Garcia W, Treviño V, Shen H, Laird PW, Levine DA, Carter SL, Getz G, Stemke-Hale K, Mills GB, Verhaak RG. Inferring tumour purity and stromal and immune cell admixture from expression data. Nat Commun. 2013;4:2612.
23. Newman AM, Liu CL, Green MR, Gentles AJ, Feng W, Xu Y, Hoang CD, Diehn M, Alizadeh AA. Robust enumeration of cell subsets from tissue expression profiles. Nat Methods. 2015 May;12(5):453-7.
24. Barbie DA, Tamayo P, Boehm JS, et al. Systematic RNA interference reveals that oncogenic KRAS-driven cancers require TBK1. Nature. 2009 Nov 5;462(7269):108-12.
25. Aran D, Hu Z, Butte AJ. xCell: digitally portraying the tissue cellular heterogeneity landscape. Genome Biol. 2017 Nov 15;18(1):220.
26. Lamb J, Crawford ED, Peck D, et al. The Connectivity Map: using gene-expression signatures to connect small molecules, genes, and disease. Science. 2006 Sep 29;313(5795):1929-35.
27. Subramanian A, Narayan R, Corsello SM, et al. A Next Generation Connectivity Map: L1000 Platform and the First 1,000,000 Profiles. Cell. 2017 Nov 30;171(6):1437-1452.e17.

Supplementary Tables

Supplementary Table S1. Characteristics of participants after imputation.

**Characteristics**	**Development cohort (N = 353)**	**Validation cohort (N = 142)**
**Age, ≥ 60 years, no. (%)**	190 (54.8)	114 (80.3)
**Male, no. (%)**	240 (68.0)	111 (78.2)
**Stage, no. (%)**		
I	173 (49.0)	25 (17.6)
II	85 (24.1)	72 (50.7)
III	90 (25.5)	35 (24.6)
IV	5 (1.4)	10 (7.0)
**Grade, no. (%)**		
G1	55 (15.6)	..
G2	170 (48.2)	..
G3	117 (33.1)	..
G4	11 (3.1)	..
**Margin residual tumor, no. (%)**		
R0	335 (94.9)	..
R1	16 (4.5)	..
R2	2 (0.6)	..
**Vascular invasion, no. (%)**	120 (34.0)	..
**Median follow-up (IQR), month**	28.0 (23.9 to 33.8)	28.5 (24.6 to 30.5)
**Median overall survival (IQR), month**	19.5 (11.2 to 26.8)	24.6 (17.7 to 35.4)
**Median disease-free survival (IQR), month**	13.1(6.5 to 25.4)	..

Notes: The variables in the development cohort with missing observation less than 20% were imputed.

Supplementary Table S2. Copper homeostasis, cuproptosis and copper binding-related genes for screening.

This table was too large to present and was presented as a separate excel file.

Supplementary Table S3. Proportional hazard assumption test for Copper-PSHC. †

End point	Cox Regression Model
Overall survival, development cohort	χ^2= 3.70, *P*= .05
Overall survival, validation cohort	χ^2=2.37, *P*= .12
Disease-free survival, development cohort	χ^2=0.49, *P*= .48

† Proportional hazard assumption test was tested with Grambsch and Therneau method.

Supplementary Table S4. The correlation between the expression of DEGs with prognostic value.

This table was too large to present and was presented as a separate excel file. The correlation between genes is represented by Spearman's coefficient at the bottom left of the table and significance was presented in terms of *P* value at the top right of the table.

Supplementary Table S5. Restricted mean survival times (RMSTs) among different Copper-PSHC risk groups and concordance index (c-index).

	Development cohort		Validation cohort	
	**High risk (N=177)**	**Low risk (N=176)**	**High risk (N=62)**	**Low risk (N=80)**
**Restricted at 3 years**				
RMST, month (95% CI)	23.9 (21.8 to 26.0)	31.3 (29.8 to 32.8)	30.9 (28.4 to 33.4)	35.0 (33.9 to 36.1)
RMST difference (95% CI) [*P* value]	-7.4 (-10.0 to -4.8) [*P* < .001]		-4.1 (-6.8 to -1.4) [*P* = .003]	
**Without time restrict**				
RMST (95% CI)	45.6 (36.9 to 54.4)	73.0 (64.1 to 81.9)	43.9 (38.9 to 48.9)	50.5 (47.2 to 53.9)
RMST difference (95% CI) [*P* value]	-27.3 (-39.8 to -14.8) [*P* < .001]		-6.6 (-12.6 to -0.6) [*P* = .03]	
**C-index (95% CI)**	0.64 (0.60 to 0.68)		0.68 (0.58 to 0.78)	

Abbreviation: RMST, restricted mean survival time; CI, confidence interval; c-index, concordance index.

Supplementary Table S6. Univariable and multivariable Cox regression of overall survival.

Variables	Univariate analysis			Multivariate analysis †		
	**HR‡**	**95% CI‡**	***P* value**	**HR‡**	**95% CI‡**	***P* value**
**Development cohort**						
**Copper-PSHC (low- vs. high-risk)**	**2.64**	**1.83 to 3.81**	**< .001**	2.33	1.60 to 3.39	**< .001**
Age (< 60 vs. ≥ 60 years)	1.24	0.88 to 1.77	.22	1.19	0.83 to 1.69	.34
Sex (Male vs. Female)	1.21	0.85 to 1.73	.29	1.11	0.77 to 1.60	.52
Grade (G1-G2 vs. G3-G4)	1.10	0.77 to 1.57	.60	-	-	-
Residual tumor (R0 vs. R1-R2)	1.94	1.01 to 3.70	**.046**	-	-	-
Vascular invasion (No vs. Yes)	1.27	0.89 to 1.81	.19	-	-	-
TNM stage (I-II vs. III-IV)	2.41	1.70 to 3.43	**< .001**	2.04	1.43 to 2.91	**< .001**
**Validation cohort**						
**Copper-PSHC (low- vs. high-risk)**	**3.30**	**1.27 to 8.60**	**.02**	3.11	1.15 to 8.42	**.026**
Age (< 60 vs. ≥ 60 years)	1.13	0.38 to 3.38	.83	1.20	0.40 to 3.61	.751
Sex (Male vs. Female)	1.43	0.51 to 4.00	.50	1.88	0.64 to 5.52	.248
TNM stage (I-II vs. III-IV)	1.92	0.79 to 4.65	.15	1.59	0.61 to 4.10	.341

Abbreviation: HR, hazard ratio; CI, confidence interval.

Notes:

† Adjusted for age, sex and stage.

‡ Hazard ratio and its 95% confidence interval were estimated by Cox regression model.

Supplementary Table S7. Subgroup analysis for Copper-PSHC of overall survival.

	HR†	95% CI†	*P* value	Event/cases
**Age < 60 years**				
Development cohort	2.33	1.34 to 4.06	.003	54/163
Validation cohort	3.26	0.46 to 23.16	.27	4/28
Merged‡	2.39	1.40 to 4.07	.001	58/191
**Age ≥ 60 years**				
Development cohort	2.79	1.71 to 4.55	<.001	75/190
Validation cohort	3.23	1.19 to 8.75	.02	16/114
Merged‡	2.87	1.85 to 4.45	<.001	237/304
**Male**				
Development cohort	3.57	2.18 to 5.85	< .001	80/240
Validation cohort	5.07	1.44 to 14.05	.005	15/111
Merged‡	3.77	2.40 to 5.94	< .001	95/351
**Female**				
Development cohort	1.68	0.95 to 2.97	.08	49/113
Validation cohort	1.24	0.20 to 7.83	.81	5/31
Merged‡	1.64	0.95 to 2.82	.076	54/144
**Stage I-II**				
Development cohort	2.07	1.30 to 3.28	.002	74/258
Validation cohort	2.50	0.71 to 8.84	.12	11/97
Merged‡	2.12	1.37 to 3.27	.001	85/355
**Stage III-IV**				
Development cohort	3.09	1.55 to 6.17	.001	55/95
Validation cohort	4.28	1.09 to 16.86	.14	9/45
Merged‡	3.73	2.01 to 6.91	< .001	64/140

Abbreviation: HR, hazard ratio; CI, confidence interval.

Notes:

† Hazard ratio and its 95% confidence interval were estimated by Log-rank test model.

‡ Hazard ratio was merged with fixed model by SAS (SAS Institute, Cary, NC).

Supplementary Table S8. Univariable and multivariable cox of disease-free survival in the development cohort.

**Variables**	**Univariate analysis**			**Multivariate analysis** †		
	**HR**‡	**95% CI**‡	***P* value**	**HR**[b]	**95% CI**[b]	***P* value**
**Copper-PSHC (low- vs. high-risk)**	**2.06**	**1.53 to 2.79**	**< .001**	**1.86**	**1.36 to 2.52**	**< .001**
Age (< 60 vs. ≥ 60 years)	1.09	0.81 to 1.46	.57	1.11	0.82 to 1.49	.50
Sex (Male vs. Female)	0.97	0.71 to 1.33	.86	0.94	0.68 to 1.29	.70
Grade (G1-G2 vs. G3-G4)	1.08	0.80 to 1.45	.63	-	-	-
**Vascular invasion (No vs. Yes)**	**1.38**	**1.02 to 1.86**	**.04**	1.23	0.91 to 1.68	.18
TNM stage (I-II vs. III-IV)	**2.19**	**1.61 to 2.98**	**< .001**	**1.85**	**1.34 to 2.56**	**< .001**

Abbreviation: HR, hazard ratio; CI, confidence interval.

Notes:

† Adjusted for age, sex and stage.

‡ Hazard ratio and its 95% confidence interval were estimated by Cox regression model.

Supplementary Table S9. Restricted mean survival times among different risk groups and concordance index.

	Development cohort		Validation cohort	
	**High risk (N=177)**	**Low risk (N=176)**	**High risk (N=62)**	**Low risk (N=80)**
**Restricted at 3 year**				
RMST, month (95% CI)	19.2 (16.4 to 22.0)	31.0 (29.7 to 32.3)	30.4 (27.2 to 33.7)	34.4 (33.3 to 35.6)
RMST difference (95% CI) [*P* value]	-11.8 (-14.9 to -8.7) [*P* < .001]		-4.0 (-7.5 to -0.5) [*P* = .03]	
**Without time restrict**				
RMST, month (95% CI)	28.0 (20.3 to 35.6)	74.7 (65.9 to 83.6)	49.7 (41.6 to 57.8)	56.7 (51.7 to 61.6)
RMST difference (95% CI) [*P* value]	-46.8 (-58.5 to -35.1) [*P* < .001]		-7.0 (-16.4 to 2.5) [*P* = .15]	
**C-index (95% CI)**	0.68 (0.63-0.72)		0.65 (0.53-0.77)	

Abbreviation: RMST, restricted mean survival time; CI, confidence interval; c-index, concordance index.

Supplementary Figures

Supplementary Figure S1. Flowchart of inclusion and exclusion of patients.

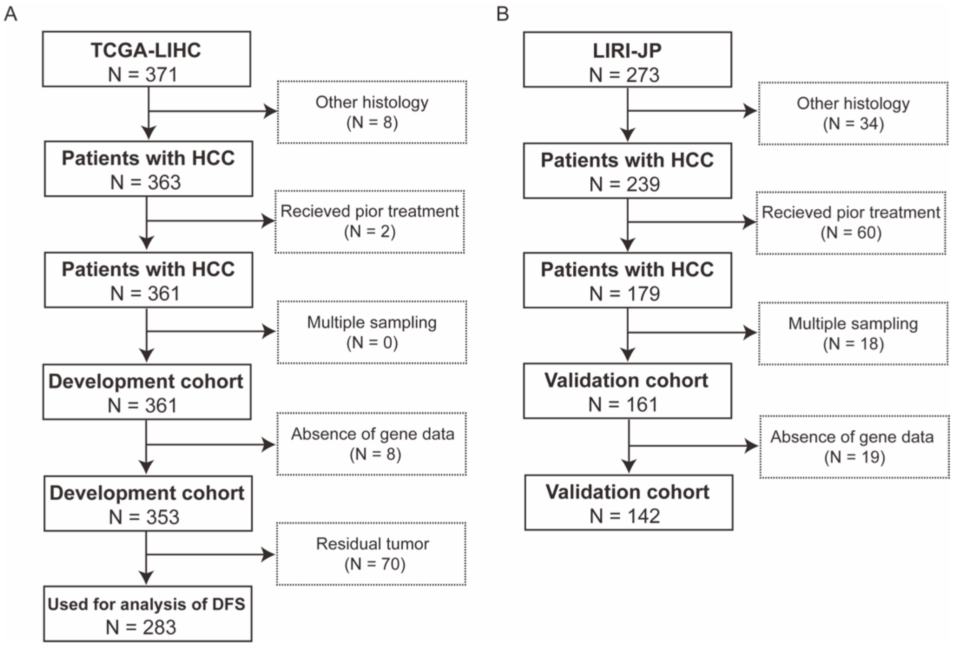

Abbreviations: TCGA, The Cancer Genome Atlas; HCC, hepatocellular carcinoma; LIRI-JP, liver cancer-RIKEN, JP; DFS, disease-free survival.

Supplementary Figure S2. Identification of the candidate copper-related genes in development cohort. **(A)** The heatmap of 19 DEGs with prognostic value between tumor and adjacent normal tissue. **(B)** Venn diagram to identify DEGs that were associated with OS. **(C)** Association of 19 DEGs with prognostic value with the overall survival. **(D)** The correlation of the expression of 19 DEGs with prognostic value. **(E)** The PPI network from the STRING database of 19 DEGs with prognostic value.

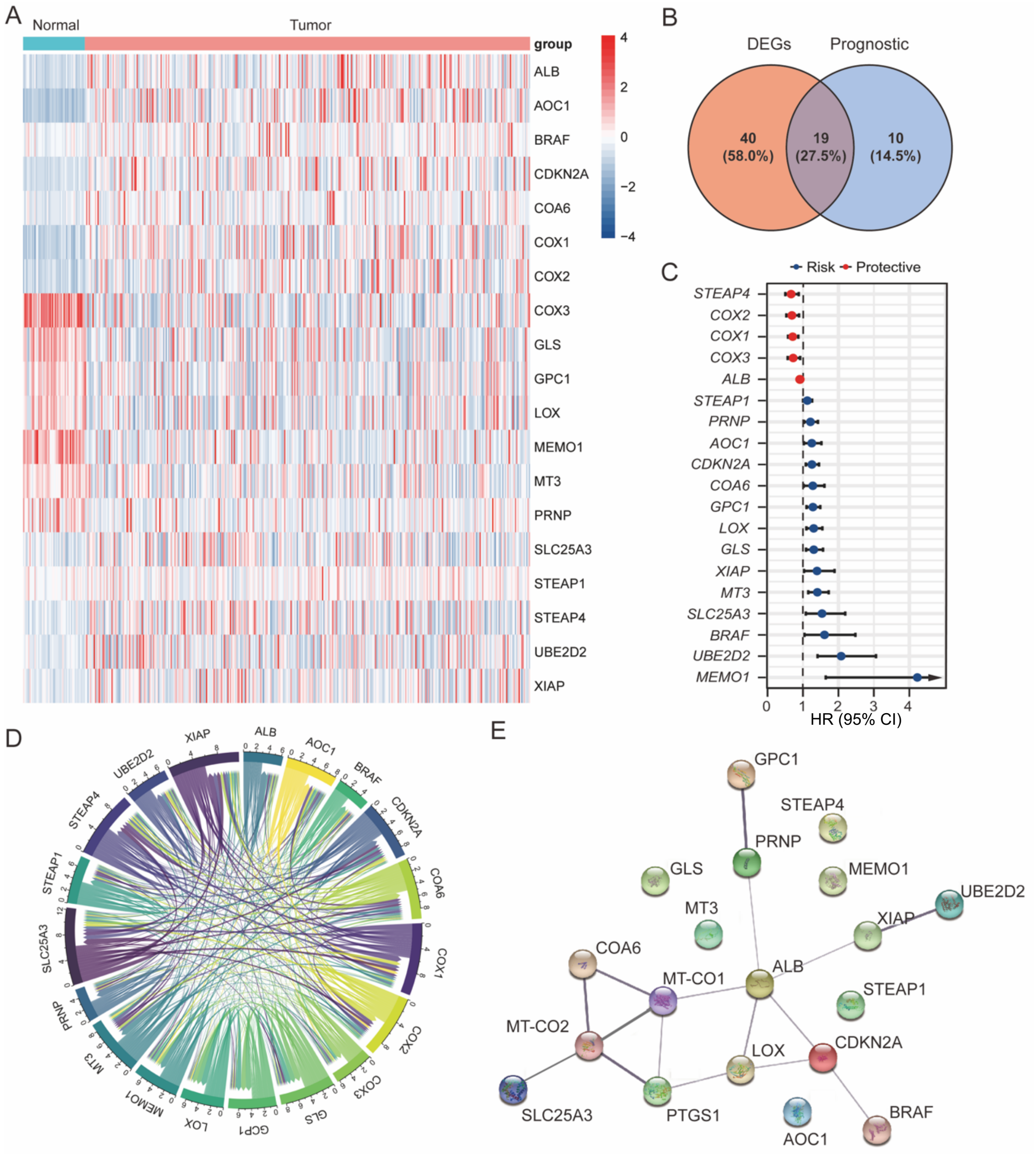

Supplementary Figure S3. The Lasso-penalized Cox analysis. The optimal penalty parameter, λ, was determined by the smallest partial likelihood deviance.

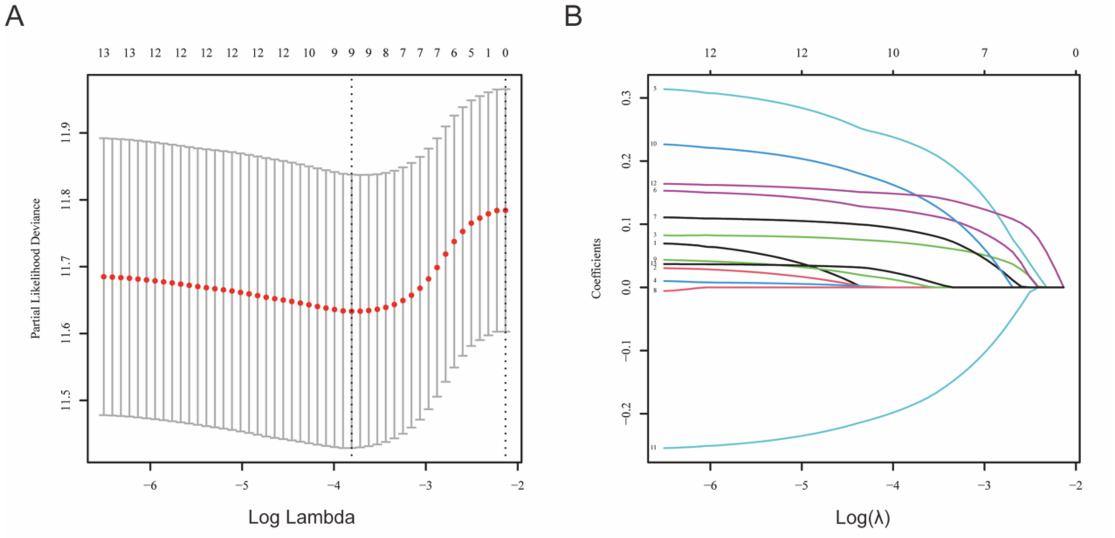

Supplementary Figure S4. Kaplan-Meier survival curves showing the association between OS and candidate genes. **(A)** *CDKN2A*, **(B)** *GPC1*, **(C)** *LOX*, **(D)** *MEMO1*, **(E)** *SLC25A3*, **(F)** *STEAP1*, **(G)** *STEAP4*, **(H)** *UBE2D2*, and **(I)** *XIAP*. For each gene, the optimal cutoff values were determined by the best stratification performance of Kaplan-Meier analyses.

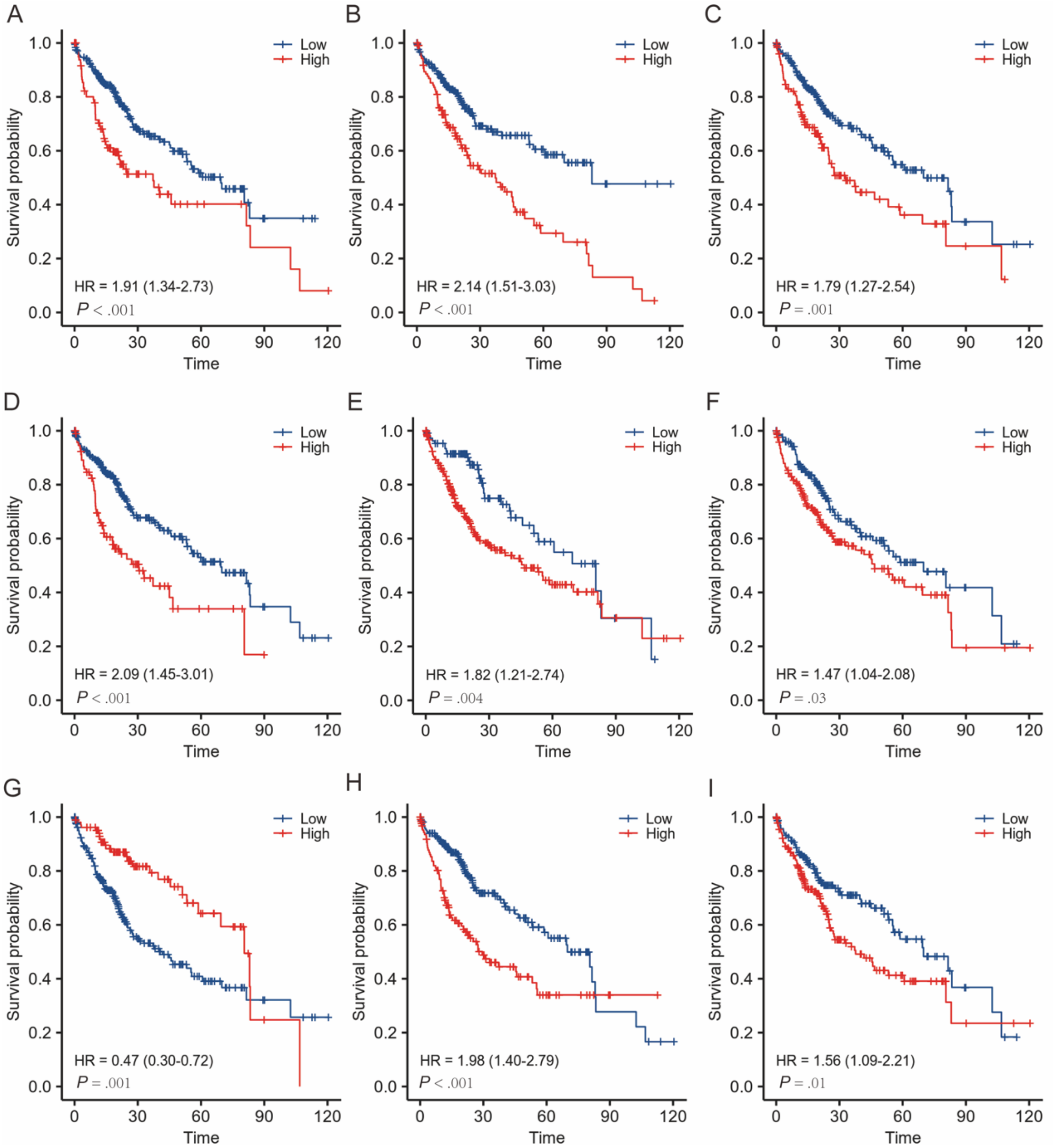

Supplementary Figure S5. Subgroup analysis of OS for Copper-PSHC in the **(A-F)** development and **(G-L)** validation cohorts. (HR1 was analyzed by Cox regression while HR2 was analyzed by log-rank test.)

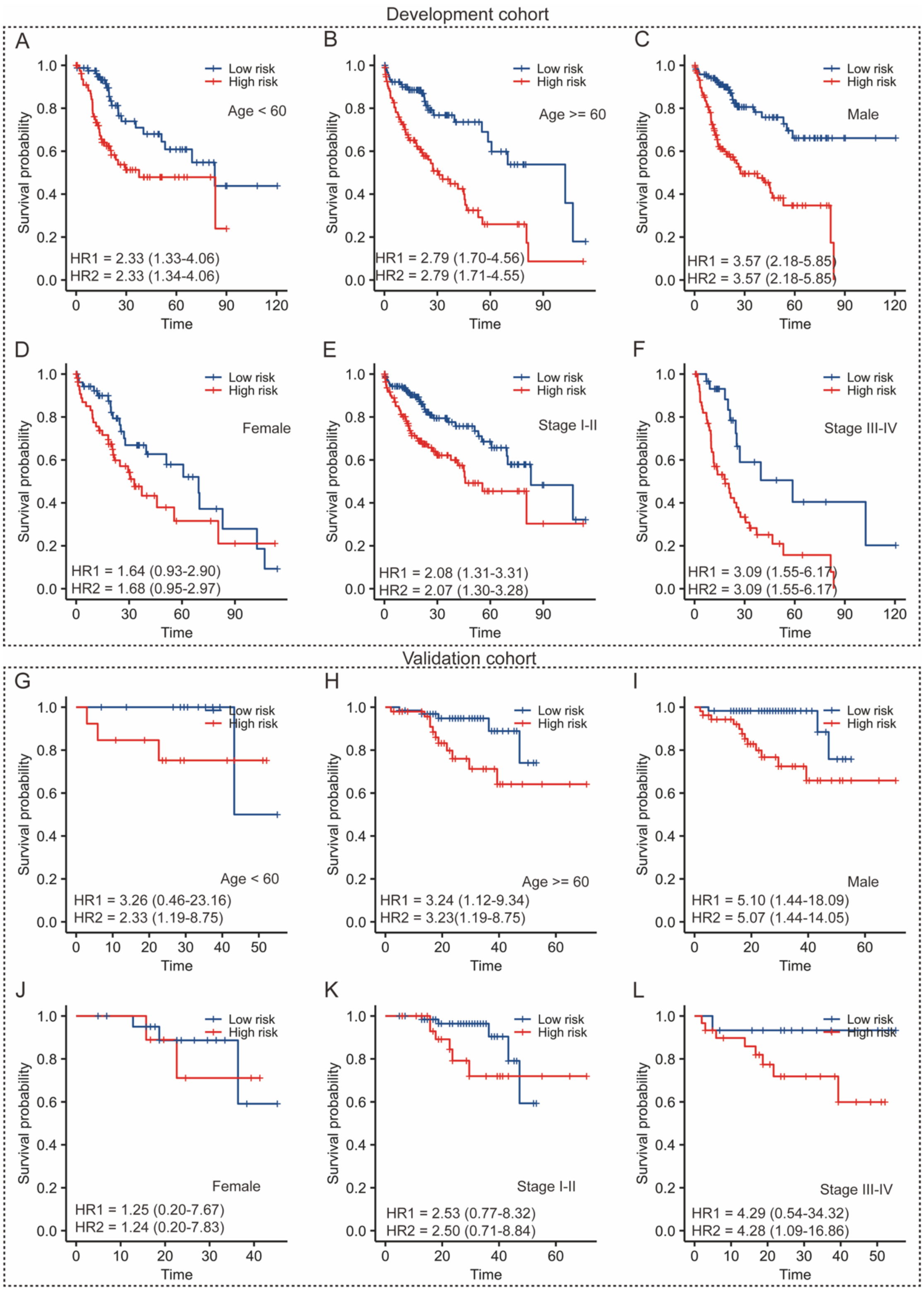

Supplementary Figure S6. Distribution of Copper-PSHC risk score among different subpopulations in the **(A-F)** development cohort and **(G-I)** validation cohort. PCA analysis in the **(J)** development and **(K)** validation cohorts. (where $*P < 0.05$, $**P < 0.01$, $***P < 0.001$)

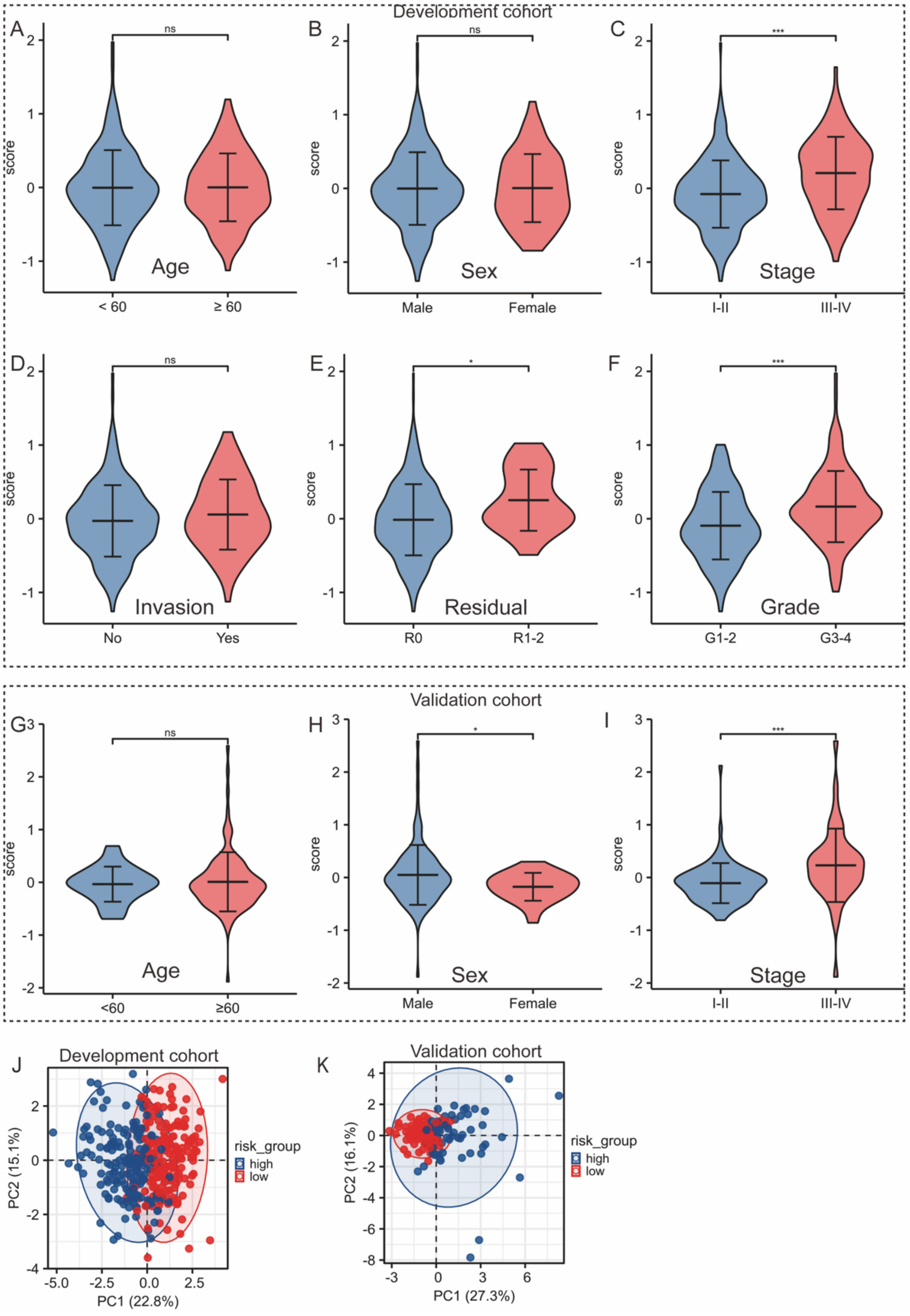

Supplementary Figure S7. Performance of the Copper-CPSHC. **(A, C)** Kaplan-Meier survival curves showing the difference of OS between high- and low- risk groups in development **(A)** and validation cohorts **(C)**. **(B, D)** ROC curves and AUC in 1-year, 2-year and 3-year OS for Copper-CPSHC in development **(B)** and validation cohorts **(D)**. **(F-G)** Calibration curves of the nomogram for the prediction in development **(F)** and validation cohorts **(G)**. **(H-K)** Decision curves demonstrating the net benefits of the nomogram in comparison with TNM stage and multivariate model containing age, sex and stage.

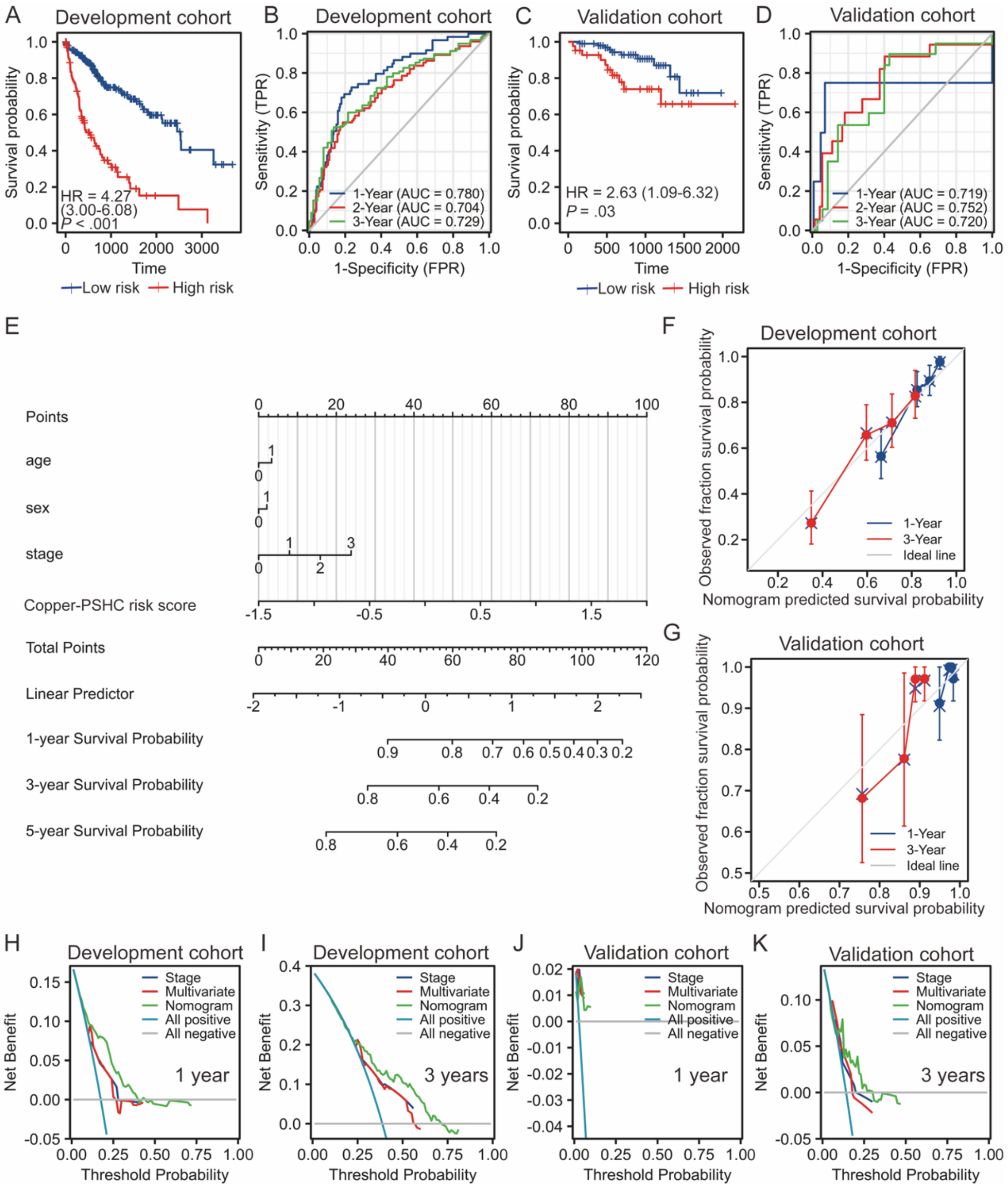

Supplementary Figure S8. Validation of Copper-CPSHC for DFS in development cohort. **(A)** Kaplan-Meier curves showing the difference of DFS in both groups. **(B)** Time-dependent ROC curves and AUC in 1-year, 2-year and 3-year DFS for Copper-CPSHC. **(C)** Calibration curves of the nomogram for the prediction of DFS. **(D-E)** Decision curves demonstrating the net benefits of the nomogram in comparison with TNM stage and multivariate model containing age, sex and stage.

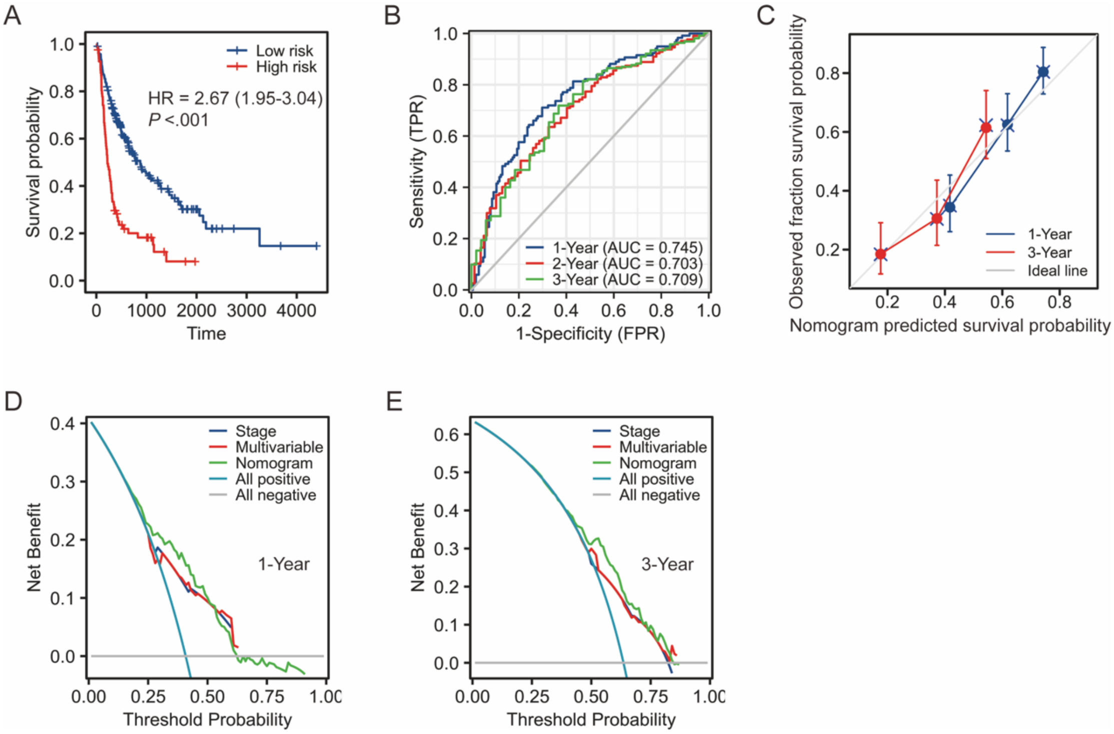

Supplementary Figure S9. Difference in tumor mutation burden (TMB) among two risk groups in the development cohort.

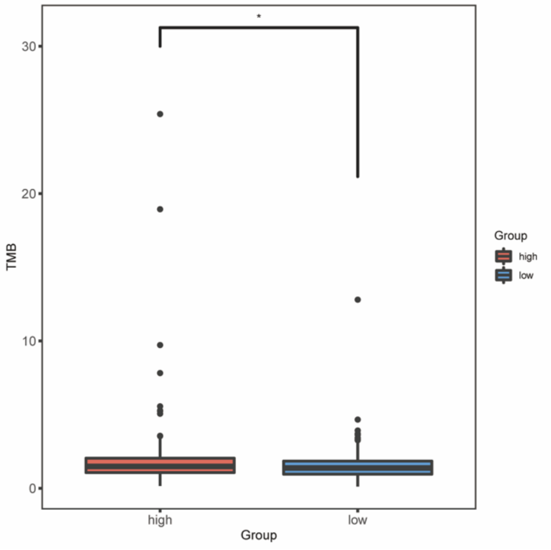

Supplementary Figure S10. Summary of mutated genes in the development for (A) low-risk and (B) high-risk group.

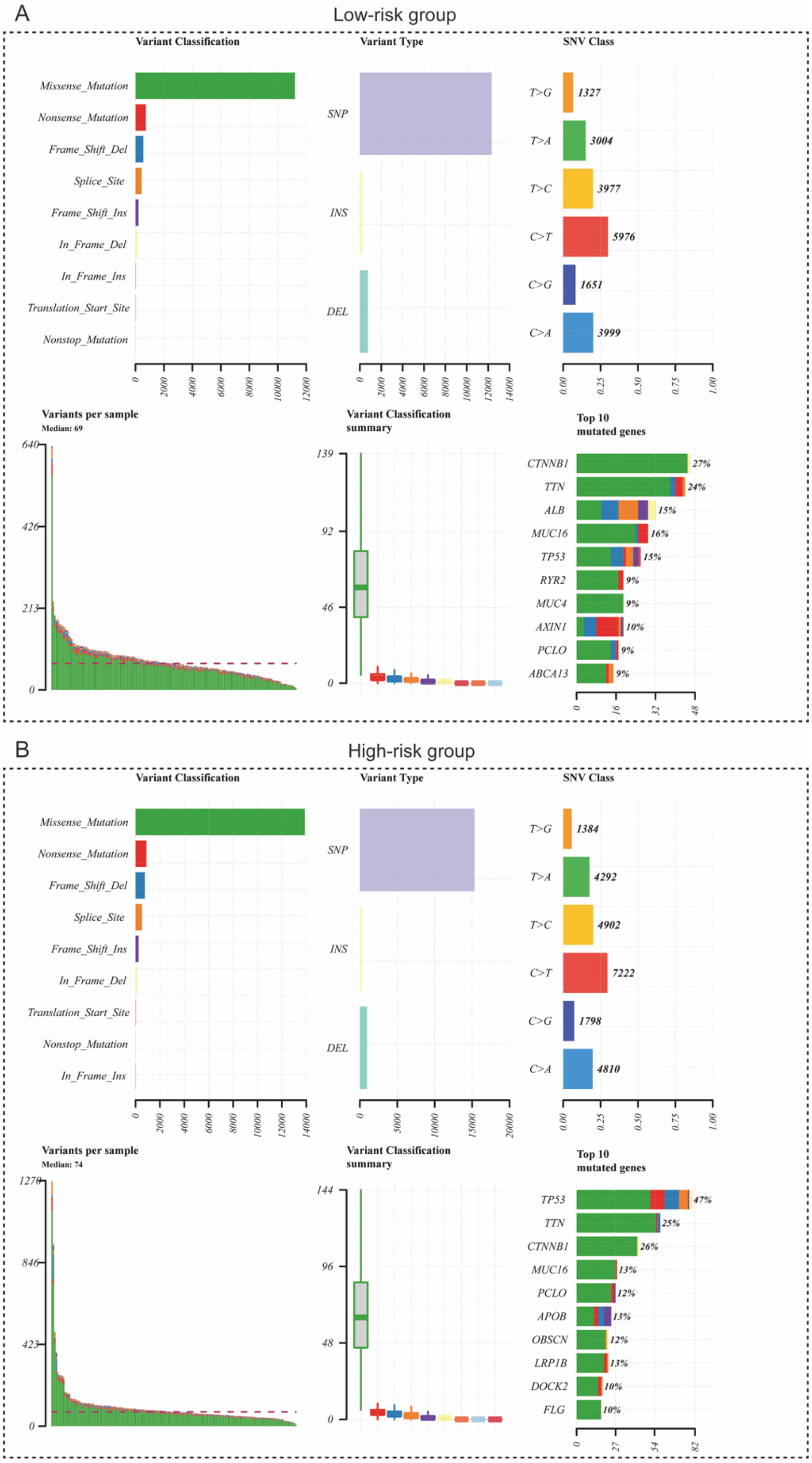

Supplementary Figure S11. Difference in hypoxia score among two risk groups in the development cohort.

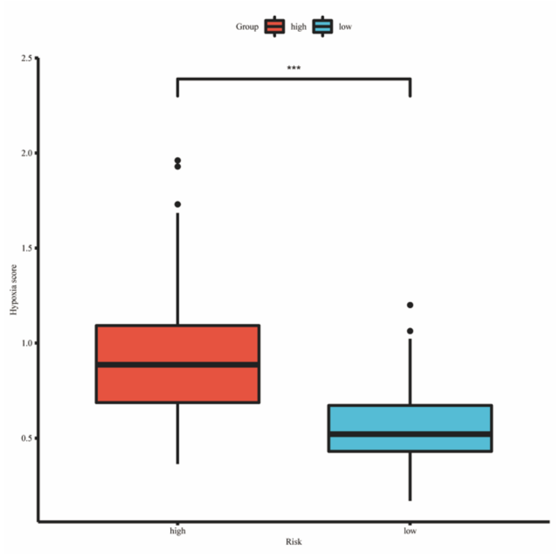

Supplementary Figure S12. Tumor microenvironment analysis in the validation cohort. **(A)** The immune, stroma and Estimate score according to ESTIMATE algorithm. **(B-D)** The differences of TME cells infiltration between high- and low-risk groups according to **(B)** Cibersort, **(C)** xCELL and **(D)** ssGSEA.

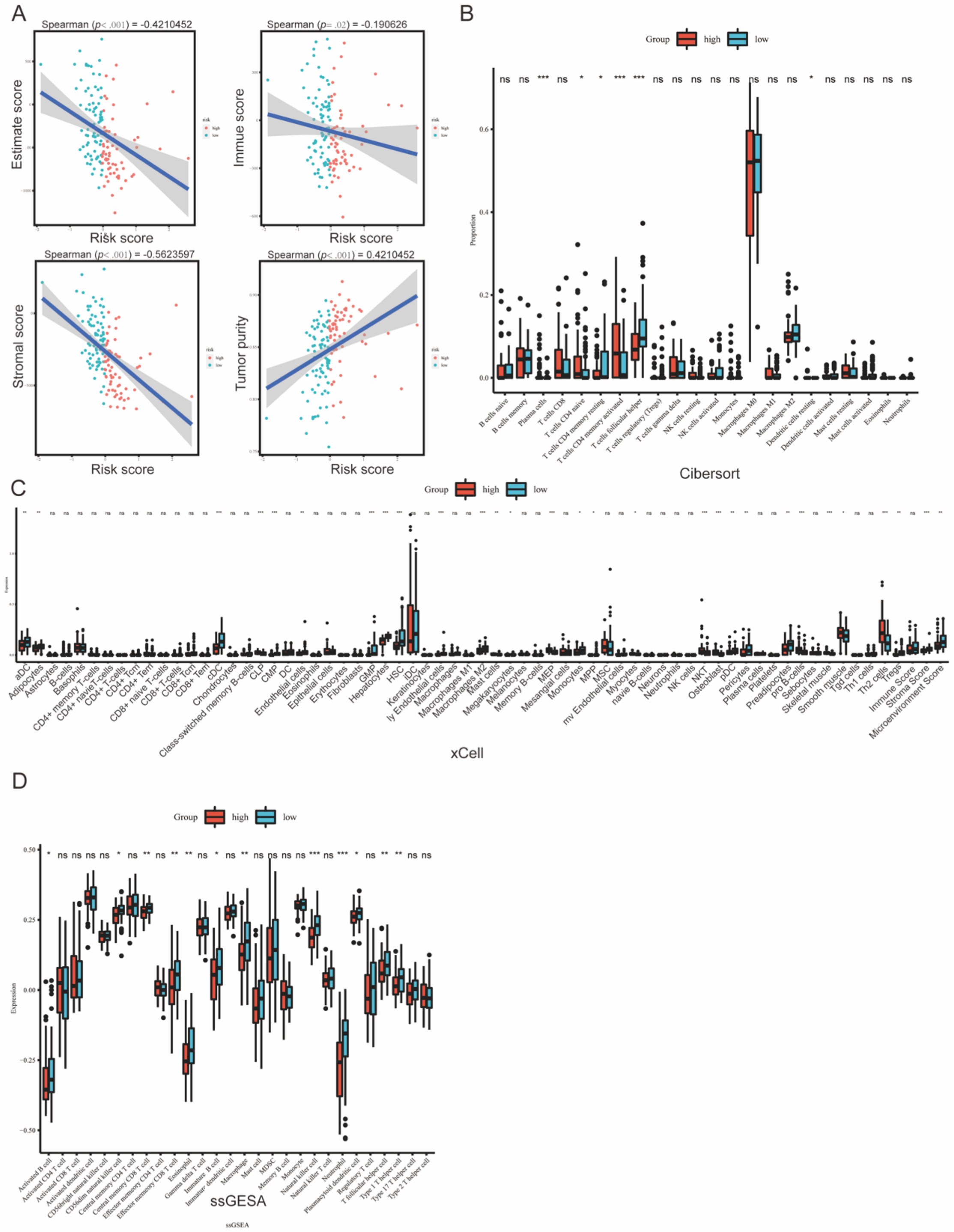

Supplementary Figure S13. The expression of cell markers related to (A) exhausted T cell and (B) ICB in the development cohort.

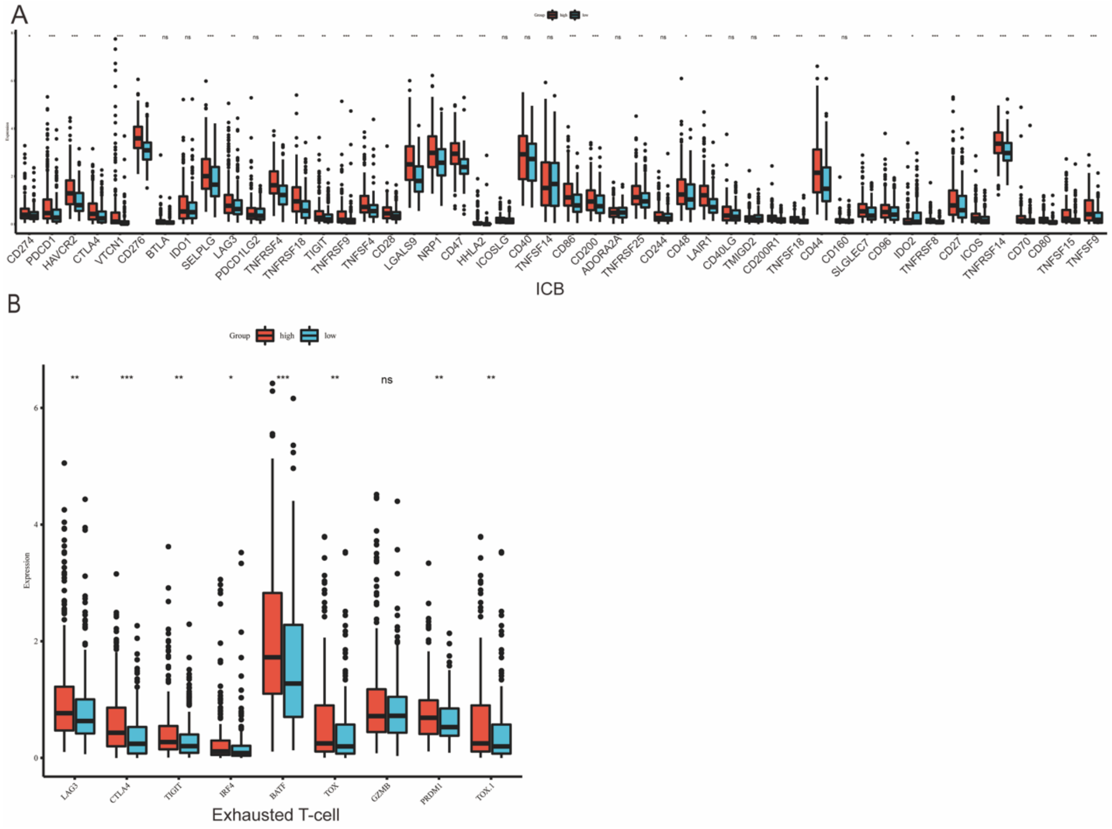

Supplement: Supplementary file 1 [file Presentation1.pdf]
